# Interferons and Interferon Regulatory Factors in Malaria

**DOI:** 10.1155/2014/243713

**Published:** 2014-07-15

**Authors:** Sin Yee Gun, Carla Claser, Kevin Shyong Wei Tan, Laurent Rénia

**Affiliations:** ^1^Singapore Immunology Network, Agency for Science, Technology and Research (A^∗^STAR), Singapore 138648; ^2^Department of Microbiology, Yong Loo Lin School of Medicine, National University of Singapore, Singapore 119228

## Abstract

Malaria is one of the most serious infectious diseases in humans and responsible for approximately 500 million clinical cases and 500 thousand deaths annually. Acquired adaptive immune responses control parasite replication and infection-induced pathologies. Most infections are clinically silent which reflects on the ability of adaptive immune mechanisms to prevent the disease. However, a minority of these can become severe and life-threatening, manifesting a range of overlapping syndromes of complex origins which could be induced by uncontrolled immune responses. Major players of the innate and adaptive responses are interferons. Here, we review their roles and the signaling pathways involved in their production and protection against infection and induced immunopathologies.

## 1. Introduction

Malaria, a mosquito-borne infectious disease transmitted by* Anopheles *mosquito, remains as one of the leading causes of morbidity and mortality worldwide, particularly in Africa, South-East Asia, and parts of South America [1]. When infected mosquito feeds on a human, the infective form of the* Plasmodium *parasite, sporozoites, is inoculated into the dermis of the host. Most of the motile sporozoites then leave the skin, travel through the blood circulation, and settle in the hepatocytes. During this liver phase, sporozoites undergo several asexual multiplications to form merozoites. Vesicles containing mature merozoites, merosomes, are released into the peripheral blood circulation and ruptured in the lungs to release thousands of merozoites into the blood circulation. These parasites infect red blood cells and initiate the erythrocytic phase [2]. Due to the exponential growth of the parasite, followed by massive destruction of erythrocytes, this stage is responsible for the common clinical manifestations of malaria such as fever, headaches, chills, and diaphoresis [3]. Usually the host immune response can control and eliminate the parasite, yet in some circumstance, patient's conditions deteriorate and develop severe systemic or organ-related pathological conditions such as anemia [4], hypoglycemia, febrile illness, respiratory distress [5], or cerebral malaria (CM) [6].

## 2. Innate Immunity to Pathogens

For the past decades, it was shown that the host immune response plays an important role in controlling the progression of malaria infection. The adaptive immunity, developed through repetitive infections during childhood, is pivotal in the elimination of* Plasmodium* parasite [7–10]. Yet, studies suggest that the host's ability to control the growth of parasites also relies on the innate immunity [11, 12]. Recent analysis of clinical records from neurosyphilis patients who underwent malaria therapy showed a controlled parasite density, irrespective of parasite strain, during the first and the second parasite inoculation which suggested the presence of a stable innate response [13]. In addition, peripheral blood mononuclear cells (PBMCs) from patients who had no prior exposure to malaria were able to produce proinflammatory cytokines, such as TNF-*α*, IL-12, and IFN-*γ*, within 10 hours of exposure to infected red blood cells (iRBCs) [14] demonstrating the activation of innate immune response against malaria parasite. However, proinflammatory cytokines are a double-edged sword. Under normal circumstances, they are essential for the control of parasite growth and sustained protection against the disease pathology, yet excessive and dysregulated production can lead to several immunopathologies [15, 16].

Human genetic diversity, parasite variability, and immune status of host generate various disease profiles of malaria infections. Fortunately, only a fraction of malaria infection in human leads to pathologies [17]. This diversity in phenotypes is always associated with differences in measured biological and immune parameters. In addition, due to obvious ethnical reasons, analyses of these parameters are largely confined to peripheral blood (serum, plasma, and circulating cells). In most studies, only association but not causal mechanisms can be determined. Thus, malaria research mainly relies on mouse models to investigate the host immune response during malaria infection. Although these models cannot reflect all aspects of human infections, they allow the study of controlled experimental infections. There are 4 rodent malaria species,* P. berghei*,* P. chabaudi*,* P. vinckei*, and* P. yoelii*, 13 subspecies, and various strains and cloned lines [18]. These parasites were isolated from African thicket rats in Central and West Africa more than 50 years ago [19]. Depending on the host and parasite combinations, different disease profiles can be induced and host immune response will determine the outcome of infection (Table 1). These models, when used together with genetically deficient mice, allow in-depth study on protection against infection or immunopathogenesis. For example, the study of CM is hampered by the limited access to tissue samples and difficulty to perform* in vivo* experiments. Susceptible mice infected with* P. berghei *ANKA (PbA) manifest neurological abnormalities similar to human CM. In this model, termed experimental cerebral malaria (ECM), high production of proinflammatory cytokines, sequestration of parasite [20–23] and leukocytes, in particular CD8^+^ T cells [24–26], and presentation of parasite antigen by brain microvessels [27] lead to the damage of the blood-brain barrier (BBB) and death. However, the role of innate immune responses in this pathology still remains to be determined.

When pathogens breach the skin or mucosal barriers, innate immune cells such as macrophages, mast cells, dendritic cells, and fibroblast, as well as circulating leukocytes, including monocytes and neutrophils, sense foreign agent using pattern recognition receptors (PRRs) that identify conserved pathogen-associated molecular patterns (PAMPs) on pathogens [28–30]. PRRs are either membrane-bound, such as toll-like receptors (TLRs) [28, 31–33] and C-type lectin receptors (CLRs) [28, 34–36], or free in the cytosol, such as NOD-like receptors (NLRs) [37–39] and RIG-I-like receptors (RLRs) [32, 40]. These PRRs are distinctly expressed on different cell populations which in turn influence the immunological repertoire elicited by a particular antigen. Professional antigen presenting cells, such as macrophage, B cells [41, 42], and dendritic cells [41, 43, 44], are well equipped with a wide spectrum of PRRs which enables this surveillance team to recognize a great variety of PAMPs and induce specific responses against each class of pathogens. For instance, in human, myeloid dendritic cells (mDCs) express all TLR1-10, but not TLR7, whereas plasmacytoid dendritic cells (pDCs) exclusively express TLR7 and TLR9 [41, 43, 44]. When activated, mDCs preferentially induces IL-12 while pDCs mainly produces IFN-*α* [44]. Other PRRs, such as dendritic cell-specific intracellular molecule-3-grabbing nonintegrin (DC-SIGN) [45] and DNGR-1 (Clec9A) [46], members of CLRs, expressed on immature DC were implicated in tolerogenic responses in some studies [47–49]. Besides professional antigen presenting cells, some epithelial cells are also furnished with PRRs. TLR2, TLR4, and TLR5 are widely found on pulmonary [50–53] and intestinal [54–56] epithelial cells. Since these surfaces are in continuous exposure to microbial challenges, strategic expression of these TLRs on these surfaces enables prompt recognition and response against bacterial infection. Vascular endothelial cells that line the entire circulatory system express also TLR4 [57, 58], RIG-I [59], and NOD-1 [60].

Upon positive PAMPs recognition, PRRs trigger a cascade of downstream signaling pathways that leads to nuclear translocation of transcription factors such as nuclear factor kappa-light-chain-enhancer of activated B cells (NF-*κ*B), activating protein-1 (AP-1), and interferon regulatory factors (IRFs) into the nucleus. These transcription factors modulate the production of inflammatory cytokines, chemokines, type I interferon (IFN-I), and some interferon-stimulated genes (ISGs) [127, 128], which in turn mobilize immune cells to target pathogens and eliminate infections. Most of these mechanisms have been identified for viral or bacterial infections [129, 130]. However, the precise mechanism by which the innate immune receptors and their signaling trigger the systemic inflammation and immune cells trafficking during malaria infection has yet to be fully uncovered. Here, we review the knowledge of the role of TLR-dependent and TLR-independent pathways and the modulation of IRFs in the activation of interferons (IFN) during malaria infection.

## 3. Recognition of Malarial Ligand by Host Receptors

Malaria parasite travels undetected in the circulation as it is encapsulated in the red blood cells. However, rupture of the matured forms of infected red blood cells exposes the parasite and releases malarial products which trigger host immune response [131–133]. This is evident by the paroxysms of fever and chills which coincide with the time of schizonts rupture [134]. The asexual erythrocytic stage of* Plasmodium* life cycle begins when merozoites are released from infected hepatocytes into the circulation. These merozoites infect red blood cells for source of nutrients and possibly also as a form of sanctuary from peripheral immune cells. Invasion is initiated by the initial contact of the parasite with red blood cells. Weak interactions of some glycosylphosphatidylinositol membrane anchors (GPIs) on the surface of merozoites [135] with receptors on red blood cells [136] trigger mechanisms that further commit the parasite to invasion [137, 138]. During invasion, most GPIs are shed from coat to facilitate entry into the target cells [139, 140]. As the parasite multiples and feeds on erythrocyte hemoglobin, it detoxifies hemoglobin heme by-product into Hemozoin (Hz) which is kept in the digestive vacuole (DV) [141–143]. Eventually, this DV, together with leftover host hemoglobin, is discharged into the circulation during egress of infective merozoites at the late schizonts stage in an explosive manner  [138, 144]. Throughout this process of invasion and egress, the* Plasmodium* parasite continually scatters malarial products which could trigger the immune system.

Extensive research has identified a few host receptors agonists from* Plasmodium *parasite which promote proinflammatory responses [61, 85, 99, 100, 102–106, 111, 112]. For the liver stage of the infection,* Plasmodium *RNA is the only malarial ligand discovered so far [92]. In the blood stage, several ligands have been identified, such as GPI [62, 99–103], Hz [63, 104, 145], CpG DNA bound on Hz [105], host fibrinogen [106], heme [107, 108], microparticles [109], AT-rich motifs in malarial genome [61],* Plasmodium *DNA/RNA [85],* P. falciparum *tyrosyl-tRNA synthetase (*Pf*TyrRS) [111], and* P. falciparum *high mobility group box protein (P*f*HMGB) [112]. All the different malarial ligands and their respective signaling molecules involved to induce an immune response are listed in Table 2. However, the exact roles of each of these factors remain to be established.

### 3.1. TLR-Dependent Signaling

TLRs are central in the sensing and responding to pathogens during innate immunity. Members of TLRs were originally identified in embryo of* Drosophila melanogaster* more than 20 years ago [146]. Later, Medzhitov et al. reported the first human homolog of the* Drosophila* toll protein that is involved in the activation of adaptive immunity [147]. To date, ten TLRs have been identified in human and twelve in mice [148]. In both human and mouse, TLRs 1, 2, 4, 5, and 6 are expressed on cell surface whereas TLRs 3, 7, 8, and 9 are found within the endosomal compartments. TLR10 is uniquely expressed in human [149] and localized on the surface of plasma membrane. TLRs 11, 12, and 13 are only functionally expressed in mice and expressed on the membrane of endosomes [150]. These TLRs recognize PAMPs ranging from DNA and RNA to bacterial products [151]. Subcellular localization of TLR ensures that different pathogenic antigens are promptly recognized by the correct receptor in order to induce proper immune responses and, at the same time, minimize accidental trigger of an autoimmune response. Upon ligand-receptor interactions, TLR signal transduction is initiated leading to production of interferons and induction of proinflammatory cytokines [31, 148, 151, 152].

#### 3.1.1. TLR Polymorphism and Malaria

Studies on genetic epidemiology revealed that TLR polymorphism is associated with outcome of malaria infection (Table 3). A population study in the Amazonian region of Brazil demonstrated that single nucleotide polymorphisms in TLR1 and TLR6 are associated with incidence of mild malaria [114]. Genetic variations in TLR1 are also capable of influencing susceptibility to placental malaria in Ghanaian mothers [113]. Case control studies demonstrated that common polymorphism in TLR2 and TLR4 can affect CM development [115, 119]. Variants in TLR2 amongst uncomplicated malaria children in Uganda were associated with altered proinflammatory responses [115] and a particular single nucleotide polymorphism in TLR4 amongst African children is correlated with an altered responsiveness to the malarial ligand, GPI, which in turn determine risk to severe malaria [119]. On the other hand, another TLR4 variant assessed in Iran (Baluchi) [116], Burundi [117], Brazil [114], and Ghana [118] was not found to be involved in malaria infection or disease severity.

Effects of TLR9 polymorphism in malaria infection have been most extensively studied amongst all the TLRs. Human genetic studies in endemic regions found a strong correlation in most of TLR9 variants with parasite load in the peripheral circulation [114, 120]. However, association of TLR9 alleles with susceptibility to malaria infection and disease severity varies according to the single nucleotide polymorphism and the regions studied [114, 116–121]. For example, TLR9 T1237C rs5743836 was associated with susceptibility to malaria infection amongst people in Burundi but not in Ghana or Iran. And amongst Ghanaians, susceptibility to mild malaria was correlated with TLR9 T1486C rs187084, but not with TLR9 G2848A rs352140.

Besides TLR, effects of single nucleotide polymorphism of coadaptor molecule, TIR domain-containing adaptor protein, TIRAP, on malaria infection were also investigated. A particular TIRAP variant was correlated with mild malaria amongst people living in Iran [116], Gambia, Vietnam, and Kenya [122]. However, when the same TIRAP alleles were sampled in Burundi and Amazon, no association with susceptibility to malaria or disease severity was observed [114, 117]. These findings suggest that variants in TLR are capable of altering disease outcome during malaria infection but polymorphism in the strains of* Plasmodium* in different regions could also account for the different association.

#### 3.1.2. TLR in Malaria Infection

Purified GPI from* P. falciparum* iRBCs [153] was preferentially recognized by TLR2/TLR1 or TLR2/TLR6 heterodimer and, to a lesser extent, TLR4* in vitro* [99, 101]. TLRs-GPI interactions trigger the recruitment of MyD88, which phosphorylates a series of mitogen-activated protein kinases (MAPKs) including extracellular-signal-regulated kinases 1/2 (ERK1/2), p38 MAPK, and c-Jun N-terminal kinases 1/2 (JNK1/2) [62, 100, 101]. Following that, nuclear translocations of transcription factor such as NF-*κ*B and AP-1, comprising the activation of transcription factor-2/c-Jun (ATF-2/c-Jun) [100, 102], stimulate production of proinflammatory cytokines such as TNF-*α*, IL-6, IL-12, and nitric oxide (NO) [100, 102, 154]. Interaction of nuclear factor of kappa light polypeptide gene enhancer in B cells inhibitor, zeta (I*κ*B-*ζ*) with NF-kB, promotes IL-12 production [103]. However, the concentration of GPI on the surface of merozoites is too low to account for this potent stimulatory effect observed [155].

A study by Pichyangkul et al. described an unknown, heat labile, malarial product in the schizont-soluble fraction that is able to upregulate expression of CD86 and stimulate IFN-*α* production by human plasmacytoid dendritic cells. When mouse bone marrow-derived dendritic cells (BMDDC) were stimulated with the same schizont fraction, upregulated expressions of CD40, CD86, and IL-12 production were observed [156]. Later, this ligand was proposed to be a metabolic by-product, Hemozoin (Hz), that is present in* P. falciparum *schizont lysate [157]. It is recognized by TLR9 to induce production of proinflammatory cytokines such as TNF-*α*, IL-12p40, MCP-1, and IL-6 [104]. However, this discovery was refuted by Parroche et al. who suggested that Hz only serves as a vehicle to deliver the malarial DNA, a TLR9 ligand, to the endosome for TLR9 sensing [105]. Similarly, Barrera et al. also supported the claim that Hz is only a vehicle for other malarial ligands, such as host fibrinogen. In this case, instead of TLR9, TLR4 and CD11b/CD18-integrin on monocytes were shown to recognize these malarial ligands [106]. In response to these, Coban et al. have recently demonstrated that both DNase-treated natural Hz and synthetic Hz are recognized by TLR9 and able to elicit an immune response via MyD88 [145]. These highly discordant results are likely due to different methodologies adopted by each group to purify the malarial Hz. Parroche et al. and Sharma et al. purified free Hz biocrystals using a magnetic separator [61, 105], whereas both Barrera et al. and Coban et al. utilized different protocols that largely consist of various chemical and mechanical procedures to obtain the natural Hz [104]. Despite disagreement on the ligand that stimulates the immune response,* in vitro *studies by both Parroche et al. and Coban et al. agreed upon the importance of TLR9 in* P. falciparum* infection. During* P. falciparum *infection, activation of TLR9 mediates production of IL-12 and IFN-*γ*. These proinflammatory cytokines in turn enhance expression of TLR and prime the signaling pathway to be more sensitive to TLR agonist [158].

Heme is released into the circulation when cell-free hemoglobin, from ruptured schizonts, is oxidized by reactive oxygen species (ROS) or other free radicals present in the plasma. The prosthetic group is recognized by TLR4, along with coreceptor CD14 [107, 108]. This interaction triggers MyD88 recruitment, I*κ*B*α* degradation, ERK1/2 phosphorylation, and eventually NF-*κ*B activation. Endotoxin contamination was abolished through the use of polymyxin B, anti-TLR4/MD2, and lipid A antagonist which inhibit effect of lipopolysaccharide (LPS) [108]. A study on a population of* P. vivax*-infected Brazilians discovered a correlation between high concentrations of heme in the plasma with disease severity. This is mediated through the activation of antioxidant enzyme Cu/Zn superoxide dismutase (SOD-1) which impairs production of anti-inflammatory mediators, such as Prostaglandin E2 (PGE_2_) and TGF-*β*, by PBMCs [107]. In the same study, plasma level of proinflammatory cytokine, TNF-*α*, was found to be positively correlated with total heme and SOD-1 [107]. Heme can be detrimental in other ways such as its toxic effects on endothelial cells [159–161] and hepatocytes [162]. At the same time, it promotes survival [163], activation [164], and migration [165] of polymorph nuclear cells. Taken together, it seems to suggest that free heme in the plasma could engage in multiple signaling pathways to promote a proinflammatory immune response, which possibly exacerbates the malaria infection.

Microparticles (MPs) are submicron vesicles produced through membrane budding during immune-activation or cell death. Extensive studies revealed that MPs are capable of influencing biological functions such as expression of adhesion molecules by endothelial cells [166] and leukocyte recruitment [167]. In addition, these minute vesicles also play a part in pathology, for instance, by inducing nitric oxide synthesis [168, 169] and delivering mRNA into other cells [170, 171]. During malaria infection, MP derived from iRBCs affects disease progression by strongly inducing bone marrow-derived macrophages (BMDM) to produce TNF [109, 172].* In vitro *study revealed that MP, and not LPS contamination, engages in a TLR4-MyD88 signaling pathway to induce this immune response. Since MP could contain other parasite proteins, like GPI and Hz, in the vesicles, it was not surprising that both TLR2 and TLR9 were also found to be involved in the induction of this MP-mediated signaling pathway. In fact, synergic engagement of all these TLRs with MP and other parasite ligands stimulated a proinflammatory response stronger than that induced by iRBCs [109].

The TLR signaling pathway is also involved in the development of placental malaria [89]. Study of* P. berghei *NK65-induced placental malaria in mice showed that MyD88 is essential in the production of proinflammatory cytokines such as IL-6, IFN-*γ*, and TNF-*α*. In addition, the MyD88 pathway also affects the survival rates of pups from malaria-infected mothers [89]. Unfortunately, no specific TLR or malarial ligands were identified to account for the activation of this MyD88 signaling pathway.

#### 3.1.3. TLR in ECM

Optimal production of proinflammatory cytokines can control parasite growth but overwhelming secretion of these soluble mediators can lead to immunopathologies such as cerebral malaria. Human population studies have demonstrated that single nucleotide polymorphism in TLRs can affect susceptibility to cerebral malaria [113–121]. However, no exact mechanism can be derived from such studies. Using the murine model of ECM, specific immune responses upon TLR-ligand interactions can be studied.

TLR2/TLR4, which recognizes malarial GPI, despite playing no role in the early stage of* P. chabaudi *infection (IFN-I secretion in this model is mediated by TLR7) [110], is important in ECM. The absence of these receptors leads to an attenuated proinflammatory response and protection from ECM lethality [63, 80]. However, different model seems to display varying degrees of reliance on this signaling pathway to induce ECM. Using wild-type (WT) and TLR2-knockout (KO) or MyD88-KO in C57BL/6 background mice infected with 10^6^ fresh PbAiRBCs intraperitoneally, Coban et al. [63] showed that ECM pathogenesis totally relies on TLR2-MyD88 signaling pathway. Activation of this pathway led to sequestration of parasites and infiltration of pathogenic T cells into the brain, two important factors responsible for damaging the brain endothelial cells. On the contrary, in this model, TLR4 was shown not to be involved in ECM development. Conversely, Kordes et al. showed that WT and TLR2/4 double knockout (DKO) in C57BL/6 background mice, infected with 10^4^ fresh PbA(clone 15cy1) iRBCs intravenously, trigger a proinflammatory response that is partially dependent on TLR2/4-MyD88 signaling pathway to cause ECM [80]. Unlike blood-stage infection, intravenous inoculation of PbA (clone 15cy1) sporozoites has absolute dependence on MyD88-dependent TLR2/4 pathway to develop ECM [80]. Inconsistency in the involvement of TLR2/4 in these models could be attributed to different infection regimens or the parasite strain/clones used. In a separate study, it was revealed that heme engages with TLR4-MyD88 signaling pathway to secrete TNF-*α* in mouse peritoneal macrophages and BMDDC. In fact, heme can also engage in a TLR4-independent pathway to induce production of ROS, expression of heme oxygenase-1 (HO-1), and recruitment of neutrophils  [108]. Besides TLR2/4, TLR9 is also shown to play a role in ECM [63]. Coban et al. demonstrated that ECM pathogenesis relied on TLR9-MyD88 signaling to induce systemic proinflammatory responses and sequestration of parasite, Hz, and leukocytes in the brain [63].

In addition, TLR9 was discovered to work in synergy with TLR7 to induce IFN-I and IFN-*γ* production in mice infected with many other strains of* Plasmodium*, like* P. chabaudi*,* P. berghei NK65*,* P. berghei K173*,* P. yoelii YM *(*Py*YM),* P. yoelii 17X *(*Py*17X), and* P. vinckei petteri *infection [90, 110]. C57BL/6 mice infected with* Py17XNL *were shown to rely on TLR9-MyD88 signaling pathway to induce production of proinflammatory cytokine and increase commitment to Th1 and cytolytic activity by NK and T cells [86].

Despite all these findings that supported the involvement of TLR2/TLR4/TLR9 in ECM development, Lepenies et al. held a different opinion. Using triple TLR2/4/9 KO mice on C57BL/6 mice, intraperitoneally inoculated with PbA iRBCs that was maintained through alternate cyclic passage in* Anopheles stephensi *mosquito and BALB/c mice, they demonstrated that ECM induction is independent of TLR2/4/9 [64]. Such disparity in research findings could be due to the unique maintenance of parasite strain/clones used. Similarly, in spite of all these studies that demonstrated the importance of TLR, the study by Togbe et al. [78] casts some doubt on the importance of TLR cascade in the development of ECM. In his study, C57BL/6 mice were infected with a cloned line of PbA tagged with GFP. Results showed that deficiency in TLR did not prevent development in ECM, lungs, or liver pathology. Once again, these conflicting results emphasized the diversity of the immune response to malaria infection in different animal models [65].

### 3.2. TLR-Independent Signaling

In the erythrocytic stage, AT-rich motifs in the* P. falciparum *genome can also induce secretion of IFN-I, TNF-*α*, IL-6, and IL-15 via a TLR-independent pathway. This ligand engages in a distinct signaling pathway that involves cytoplasmic nucleic sensor STING, downstream kinase TBK1, and interferon regulatory factor (IRF) 3/7 [61]. Besides,* Pf*TyrRS [111] and P*f*HMGB [112] are two other malarial ligands that were shown to induce proinflammatory activity. However, more studies are needed to identify the specific receptors that recognize these two ligands. Other cytoplasmic signaling molecules, such as the member of RLR family and its adaptor molecule, melanoma differentiation-associated protein 5/mitochondrial antiviral signaling protein (MDA5/MAVS), were also associated with IFN-I signaling during acute phase of nonlethal* P. yoelii nigeriensis* N67 (*PyN *N67) infection in C57BL/6 mice [85]. On the contrary, STING and MAVS were found to be redundant in early* P. chabaudi* infection [110] and* P. yoelii *liver-stage infection [126].

Host innate immune response does not only exist in the erythrocytic stage of the parasite life cycle. In fact, control of parasitic growth starts as early as in the asymptomatic liver stage [173, 174]. Liver resident cells of C57BL/6 and BALB/c mice were shown to induce IFN-I production upon infection with PbA or* Py*17XNL sporozoite, independent of TLR-MyD88 pathway [92]. The* Plasmodium *RNA was found to be identified by MDA5, which typically recognizes double stranded DNA. This triggers the assembly of MAVS, which mediates downstream production of IFN-I. Liver-stage specific IFN-I mobilizes leukocytes into the vicinity of infected hepatocytes to limit parasite load in the liver and consequently influences the induction of erythrocytic stage infection. Besides MDA5, there are other unknown malarial ligands that signal through Mavs as evident by the differential IFN-I response in MDA5^−/−^ and MAVS^−/−^ mice [92].

## 4. Interferons

Recognition of PAMPs by PRRs triggers a cascade of downstream signaling pathways which stimulates production of IFN-I, IFN-*γ*, and many other proinflammatory mediators. The IFN compartment comprises 3 classes, namely, IFN-I, IFN-II, and IFN-III. IFN are renowned for their antiviral properties and share common secondary structure. Yet each class of IFN binds to distinct multichain receptor complexes. They engage in different JAK-STAT molecules, drive the expression of different interferon stimulated response elements (ISRE) and/or interferon-gamma activated sequences (GAS) elements [175], and induce various interferon stimulated genes (ISGs), which in turn regulate development, host defense, and signaling [176]. In humans and mice, the IFN-I family comprises 13 types of IFN-*α* and 1 IFN-*β*  [177]. IFN-II consists of solely interferon gamma (IFN-*γ*), while there are 3 types of IFN-III, namely, IFN*λ*1, IFN*λ*2, and IFN*λ*3 [175].

### 4.1. Type II Interferon (IFN-*γ*)

IFN-*γ* is the only form of type II IFN. It regulates several components of the immune system such as antigen presentation [178–181], antimicrobial mechanism [182–184], leukocyte development [185], and immune cells trafficking [186, 187]. It is the most widely studied interferon in malaria infection since it is primarily involved in host defense against intracellular pathogens. Its protective role as an immune mediator emerged as early as in the liver stage [126, 188–193].* In vitro *study of human recombinant IFN-*γ* treatment on* P. berghei *sporozoites-infected murine hepatocytes [190] or human hepatoma cells [189] identified an inhibitory effect of IFN-*γ* on parasite multiplication. Further* in vivo *study validated the importance of IFN-*γ* in protective immunity as it inhibits intracellular development of parasite within hepatocytes following challenge with* P. berghei *[194],* P. yoelii *[191], or* P. vivax *sporozoites [188] in mice and chimpanzee, respectively. Recently, Miller et al. demonstrated that IFN-*γ* secreted in primary* P. yoelii* sporozoite infection is the key innate mediator that controls liver-stage parasite growth in a secondary infection [126]. Above all, this inhibitory effect of IFN-*γ* on parasite development in liver stage extends and influences the initiation of blood stage parasite growth [126].

IFN-*γ* also plays a crucial protective role during blood-stage infection of various parasite strains. Administration of exogenous recombinant IFN-*γ* leads to control of parasite growth in* P. chabaudi adami *556KA*-*infected CBA/CaH mice. After infection has been resolved, continuous IFN-*γ* treatment fully protected these mice from subsequent infection [96]. Lower level of parasitemia was also observed in IFN-*γ* treated SW mice that were infected with lethal strain of* P. yoelii.* In addition, these treated mice also exhibited better survival outcome [94].* P. chabaudi *AS-infected mice treated with monoclonal antibody against IFN-*γ* had less control of parasite multiplication [195], once again suggesting that IFN-*γ* is essential for limiting parasite growth. Similar findings were observed in* P. chabaudi *AS-infected mice that were deficient in IFN-*γ* receptor. However, these mice had lower survival rates as compared to the WT controls [91]. This suggests that IFN-*γ* production at different period during infection could alter survival outcome. In* P. berghei *infection, IFN-*γ* also plays a protective role by mediating parasite clearance [196]. Population study of children in Papua New Guinea showed that high and early IFN-*γ* responses seem to protect from symptomatic malaria [197].

However, production of high level of IFN-*γ* during parasite blood stage development is associated with predisposition to severe malaria, such as CM. Studies in animal model of ECM corroborated findings from human study that IFN-*γ* is essential for the development of CM [66]. IFN-*γ* signaling in the brain regulates expression of adhesion molecules which influence parasites and leukocytes sequestration in the brain microvessels [97]. At the same time, there is also evidence that IFN-*γ* promotes trafficking of leukocytes, including pathogenic CD8^+^ T cells, to the brain [81, 83]. IFN-*γ* is essential in both protective immunity and pathogenesis of severe diseases [198]. Whether it protects or harms the host depends on when and where it is produced [67].

### 4.2. Type I Interferon (IFN-*α*/*β*)

Unlike IFN-*γ*, IFN-I is only starting to gain more attention with increasing evidence that supports its role in protection [87, 199, 200]. This cytokine regulates various immune mechanisms such as MHC expression [201], antigen presentation [201], and T cell expansion [202, 203]. Furthermore, IFN-I can also modulate production of IFN-*γ* [204] and prime IFN-*γ*-mediated immune responses [205]. The earliest report which revealed its significance in malaria demonstrated that exogenous administration of unpurified mouse serum IFN was able to protect CF-1 mice from PbA sporozoite infection [206]. Although the experiment suggested a role for IFN-*Ι*, this unpurified mouse serum contains mediators other than IFN-*Ι* [207], such as IL-6, which have activity against* Plasmodium *liver stages [208]. Following that, a study of treatment with recombinant human IFN-*α*, which cross-reacts with mouse cells, did not show any effect on near matured (42 h) liver stage after a challenge with* P. yoelii *sporozoite [87]. However, recent analysis of liver transcriptome obtained from PbA [92] or Py [92, 126] sporozoite-infected C57BL/6 or BALB/c mice revealed an upregulation of genes expressions that are linked to IFN signaling. IFN-*Ι* was found to act during the very late phase of the liver stage and this liver specific IFN-*Ι* production partially limits parasite growth in the liver and influences initiation of erythrocytic stage infection [92]. In mice,* P. yoelii *and* P. berghei* liver stages last a minimum of 48 h and 51 h, respectively [209], and the effect of IFN-*Ι* was only apparent after 48 h but not 42 h after sporozoite infection. Interestingly, the IFN-*Ι* effect was indirect and mainly mediates the recruitment of leukocytes around liver-stage parasites. IFN-*γ*-secreting immune cells, in particular CD1d-restricted NKT cells, are the main players responsible for the innate elimination of liver stage [126]. Leukocyte-mediated inhibition of liver-stage parasite further leads to a reduced development of parasitemia [92]. More importantly, this innate immune response can facilitate parasite elimination in subsequent liver-stage infection [126, 191]. In fact, early production of *Ι*FN-I prior to infection can impair parasite establishment [92].

Compared to liver-stage infection, *Ι*FN-*Ι* has a more striking role against blood stage parasites. Previous work shows that treatment of C57BL/6 mice with pure recombinant IFN-*α* inhibits* P. yoelii *or* P. berghei *blood stage development [87]. This effect was indirect and mediated by IFN-*γ* [210]. During early stage of* P. chabaudi *infection, IFN-I induced by the infection plays a pathogenic role by suppressing IFN-*γ* producing CD4^+^ T cells that control parasite load in C57BL/6 [77] but not in 129 Sv/Ev mice [98]. These results are not contradictory but suggest different levels of IFN-I and the duration of action is essential for proper immune response to control parasite growth.

In human, polymorphism in the receptor of IFN-I, IFNAR, has been shown to be robustly associated with progression of CM [79, 200]. It was further revealed that peripheral blood mononuclear cells from Malawian children recovering from severe malaria had higher expression of genes involved in interferon pathway [211]. In murine model, recombinant IFN-*α*  [210] or IFN-*β*  [68] treatment protected mice from ECM death. When PbA-infected C57BL/6J mice were administered with recombinant human IFN*α*, increased level of IFN-*γ* in treated mice was observed, which was linked to improved control of parasitemia and survival [210]. On the other hand, IFN-*β* treatment prevented ECM death by suppressing the expression of chemokine receptor CXCR3, the production of IFN-*γ*, and chemokine ligand CXCL9. Consequently, decreased T cells migrate and sequester in the brain thereby preserving a better vascular integrity of blood brain barrier as compared to nontreated WT controls [68]. However, IFN-I induced endogenously during* Plasmodium* infection plays a pathogenic role in ECM development. Absence of IFN-I signaling, in mice deficient in IFNAR, either delayed [82] or fully protected [77, 79] the mice from ECM. Haque et al. [77] attributed this protection to a restrained parasite growth in the absence of IFN-I pathway whereas Ball et al. [79] and Palomo et al. [82] concluded that deficiency in IFN-I signaling reduced sequestration of pathogenic T cells in the brain. All these conflicting data suggest that effects of IFN-I might rely on precise level and timing of expression of systemic IFN-I.

## 5. Interferon Regulatory Factors

Both IFN-I and IFN-*γ* are essential in the immune response against malaria infection. Production of IFNs is triggered upon recognition of malaria antigen by receptors as discussed above. Although an array of receptors and downstream signaling molecules have been implicated, all signaling pathways ultimately converge to a few downstream transcription factors, such as IRFs, which regulate gene expression of IFNs. The family of IRFs comprised 9 members, namely, IRF1-9. Each IRF binds to a unique set of ISRE to stimulate transcription of diverse genes that are translated into functional proteins [212, 213]. The diverse roles of a few IRFs in malaria infection have been uncovered recently (Table 4).

### 5.1. Interferon Regulatory Factor 1

The first member of the IRF family identified that binds to the promoter region of IFN-*β* gene is IRF-1. This transcription factor is expressed in many cells types. It mediates signaling of both IFN-I and particularly IFN-*γ*, a strong inducer of IRF-1 expression. IRF-1 regulates antigen presentation, monocyte/macrophage differentiation, T cell development, and B cell growth [214] and promotes Th1 response [215]. In humans, the IRF-1 gene is located in chromosome 5q31-33 region and variation in 5q31-33 region was associated with variations in parasite density during* P. falciparum *erythrocytic infection [216]. A subsequent study in West African ethnic groups identified that polymorphisms in IRF-1 gene could lead to differential abilities to control* P. falciparum *infection [123]. Despite the fact that Mangano et al. discovered a correlation between IRF-1 and control of* P. falciparum *infection [123], they found no association of this transcription factor in the development of severe malaria pathology amongst African children [124]. Using mice deficient in IRF-1, Tan et al. showed that IRF-1 is essential in limiting parasite growth and survival outcome of PbA infection [125]. Further animal studies also demonstrated that IRF-1 regulates antigen presentation [214] and is indispensable in the pathogenesis of ECM. When infected with PbA, mice deficient in IRF1 were partially protected from ECM with a lesser control in parasite growth in the circulation [69]. Microarray analysis of brains from ECM-susceptible C57BL/6 mice as compared to ECM-resistant BALB/c mice revealed an increase of IRF-1 gene expression [70]. Similarly, IRF-1 gene expression was higher in brain from CBA/T6 mice infected with ECM-causing PbA parasite than with non-ECM causing* P. berghei *K173 parasite [95]. With this evidence, there is a need to further investigate the exact implication of IRF-1 in these different immune mechanisms which are essential for ECM pathogenesis [27].

Recently, Wu et al. also demonstrated that higher expression of IRF-1 gene and production of IFN-I enabled better control of parasitemia in nonlethal* P. yoelii *N67-infected mice than in lethal* P. yoelii *N67C-infected mice [85]. However, the role of IRF-1 in IFN-*β* signaling remains controversial as stimulation of splenocytes from IRF-1 deficient mice with malarial genome-alike AT-rich oligonucleotides did not abrogate IFN-*β* production [61]. This discrepancy could be due to the use of different malaria ligand to induce IFN-I production comforting previous speculation of multiple signaling pathway to produce IFN-I [217].

### 5.2. Interferon Regulatory Factor 3/7

Among the 9 IRFs, IRF-3 and IRF-7 are the master regulators of IFN-I. They are responsible for driving the initial transcription of IFN-I during early stage of infection. Induction of IFN-I is generated through a biphasic mechanism which warrants transcriptional efficiency and diversity of targeted genes. IRF-3 is constitutively expressed in the cytoplasm of all cells and resides as an inactive form. Upon phosphorylation, activated IRF-3 translocates into the nucleus and forms enhanceosome with other transcription factors, namely, NF-kB and AP-1 [218], which will lead to IFN-*β* transcription [219–221]. On the other hand, IRF-7 is expressed at very low levels in the cytoplasm of most cells. Positive feedback of IFN-*β* increases IRF-7 expression. Like IRF-3, it undergoes nuclear translocation and forms heterodimer with IRF-3 to bind with ISRE. Unlike IRF3, IRF-7 induces maximal transcription of both IFN-*α* and IFN-*β*  [222]. The role of IRF-3 and IRF-7 in malaria infection remains poorly defined.

When mice deficient in either IRF-3 or IRF-7 were infected with PbA sporozoite, significant impairment in IFN-I response was observed. Consequently, these deficient mice had higher parasite load in the liver and peripheral circulation as compared to their WT counterparts. Initiation of blood-stage infection was also found to be 1-day earlier in the KO as compared to WT mice [92]. On the other hand, only mice deficient in IRF-3 displayed marked impairment in the control of parasite burden in the liver upon secondary* P. yoelii *sporozoite infection [126]. Such disparity could be attributed to the strain of parasite or the time point measured in each study. Since IRF-3 stimulates IFN-*β* production [219–221] and IRF-7 induces both IFN-*α* and IFN-*β* production [222], the significance of IRFs in each infection model could possibly hint on the importance of IFN-*α* and/or IFN-*β* at different window of the liver-stage infection. When stimulated with infected red blood cells or AT-rich motif derived from genome of* P. falciparum*, splenocytes obtained from mice deficient in both IRF-3 and IRF-7 had attenuated IFN-*β* production, demonstrating a role for one or both of these factors in IFN-*β* production [61]. In mice infected with* P. chabaudi*, early IFN-*α* production by red pulp macrophages is dependent on both IRF-3 and IRF-7. Intriguingly, contrary to what is observed with viruses [223–226], IFN-*β* production was independent of IRF-3 [90, 110], suggesting an alternate pathway of activation for malaria parasite. Microarray analysis of brain from ECM-susceptible mice showed a higher transcriptional activity of IRF-7 than ECM-resistant [70] and uninfected control mice [71]. Double IRF-3/IRF-7 deficient mice infected with PbA were resistant to ECM upon infection [61] confirming a role for IFN-I in ECM. However, the precise functions of IRF-3 and IRF-7 in CM remained to be determined.

### 5.3. Interferon Regulatory Factor 8

IRF-8 is one of the unique IRFs that is only expressed in immune cells [227]. Unlike IRF-3 and IRF-7, expression of IRF-8 is induced by IFN-*γ* instead of IFN-I. This transcription factor coordinates growth and differentiation of myeloid cells, such as macrophages and dendritic cells, and production of proinflammatory cytokines, such as IFN-I and IL-12p40 [227]. Together with IRF-1 [228], IRF-8 directs transcription programs in immune cells towards a Th1-dominated response [229]. Since ECM is a Th1-mediated pathology [230–232], it is not surprising that amplification in IRF-8 gene expression was observed in the brains of ECM-susceptible PbA-infected CBA/T6 mice [95]. Mice with dysfunctional IRF-8 are protected from ECM due to downregulated transcriptional activity of many IRF-8-dependent genes which are essential in various aspects of the immune response during PbA infection. These modulated genes are involved in antigen processing and presentation, chemotaxis, maturation of phagosomes, and production of proinflammatory cytokines [72]. Though both reports concurred that IRF-8 is involved in ECM development, further research is mandatory to ascertain its role in human CM.

### 5.4. Other Interferon Regulatory Factors

Apart from IRF-1, IRF-3, IRF-7, and IRF-8, some studies have also briefly explored the role of IRF-5 and IRF-9 in malaria infection. IRF-5 is expressed in B cells and dendritic cells. Like IRF-7, it is mainly regulated by IFN-I. This transcription factor interacts with IRF-1, IRF-3, and IRF-7 to induce expression of proinflammatory cytokines [233, 234]. The only report on IRF-5 in malaria infection revealed that it is dispensable in the production of IFN-*β* by splenocytes in response to stimulation by AT-rich oligonucleotides that resemble those in the malarial genome [61]. Another member, IRF-9, is expressed constitutively in many cell types and unlike the rest of the IRFs, it functions only when it dimerizes with STAT1 and STAT2 to form an active trimeric complex, known as ISGF3. This complex binds to ISRE and activates ISGs [235, 236]. During nonlethal* PyN*N67 infection, IRF-9 participates in the production of IFN-I to control parasite growth [85]. A robust IRF-8-dependent amplification of IRF-9 was detected in brain of mice infected with PbA [72]. These separate studies seem to hint on the possibility of more IRFs involvement in the immune response during malaria infection.

## 6. Future Perspectives

**Figure 1:**
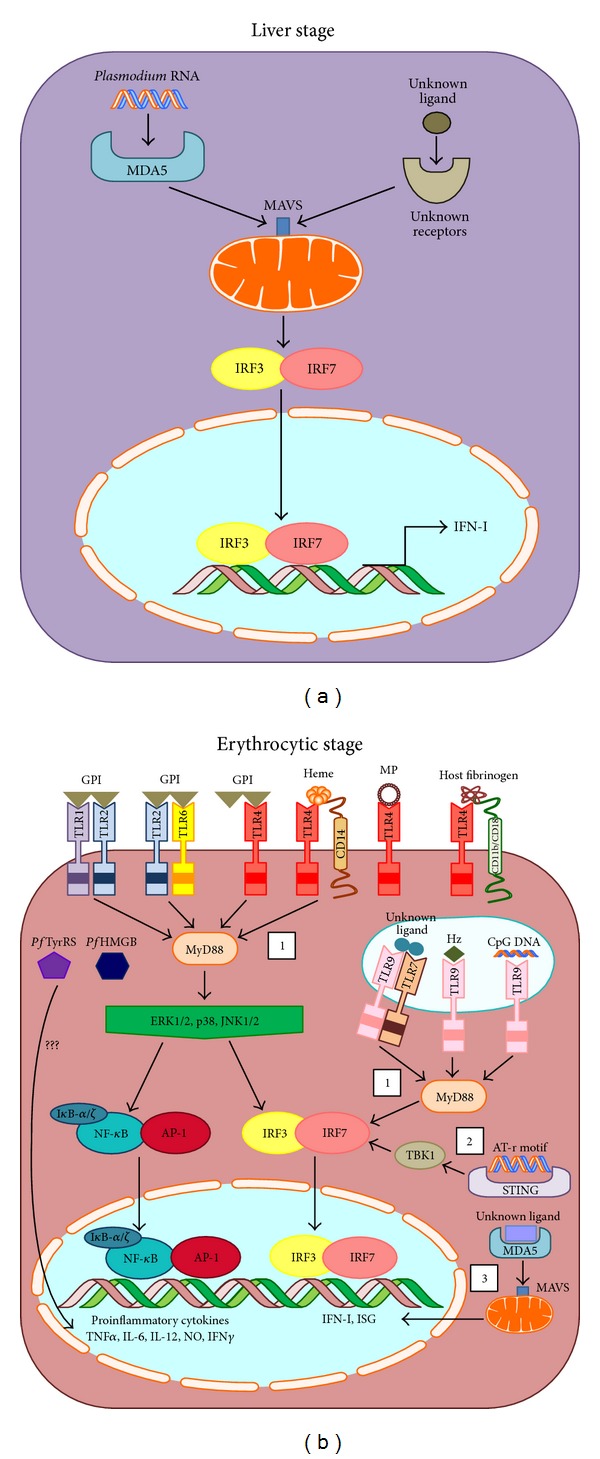
(a) Signaling pathway induced by malarial ligand during liver-stage infection.* Plasmodium *RNA is recognized by MDA5 (melanoma differentiation-association protein 5) present in the cytoplasm. Ligand-receptor interaction triggers assembly of MAVS (mitochondrial antiviral signaling protein) that aggregate on the surface of mitochondria. This eventually leads to the activation of both IRF-3 and IRF-7 which regulate transcription of IFN-I. Besides MDA5, activation of other receptors can also trigger aggregation of MAVS. However, this specific receptor and its corresponding malarial ligand have yet to be identified. (b) Signaling pathway induced by malarial ligand during erythrocytic-stage infection. Surface TLR4 recognizes a number of malarial ligands such as GPI (glycosylphosphatidylinositol membrane anchor) and MP (microparticles). Together with CD14 or CD11b/CD18 integrin, it can recognize heme and host fibrinogen, respectively. Both TLR heterodimer TLR1/TLR2 and TLR2/TLR6 recognize GPI. Within the endosomal compartment, Hz (Hemozoin) and CpG DNA are recognized by TLR9. In addition, TLR7/TLR9 heterodimer has been proposed to recognize an unknown malarial ligand. These ligand-receptor interactions trigger 3 proposed pathways. (1) TLR-dependent pathway involves the recruitment of MyD88 (myeloid differentiation primary gene 88) to TLR, which phosphorylates downstream MAPKs (mitogen-activating protein kinases), such as ERK1/2 (extracellular-signal-regulated kinases 1/2), p38 MAPK, and JNK1/2 (c-Jun N-terminal kinases 1/2). Subsequently, NF-*κ*B (nuclear factor kappa-light-chain-enhancer of activated B cells) and AP-1 (activating protein-1) translocate into the nucleus and stimulate production of proinflammatory cytokines. At the same time, phosphorylated MAPKs or MyD88 can induce activation of IRF-3 and IRF-7 to transcribe IFN-I and ISGs (interferon stimulated genes). (2) Activation of TLR-independent pathway triggered by AT-rich motif present in the* plasmodial *genome engages STING, TBK, IRF-3, and IRF-7. (3) Another TLR-independent pathway involves MDA5 and MAVS.* Pf*TyrRS (*P. falciparum *tyrosyl-tRNA synthetase) and* Pf*HMGB (*P. falciparum *high mobility group box protein) were shown to induce proinflammatory responses but the exact signaling pathways have yet to be identified.

In Figure 1, we illustrate the different malarial ligands and the various signaling pathways triggered to produce IFN-I and proinflammatory cytokines in the liver and erythrocytic stages. Though controversial, these studies demonstrated that IFN-I [77] and IFN-*γ* [94] produced during infection may modulate the course of disease progression. However, the same immune response that initially protects the host could inevitably contribute to the pathogenesis of severe malaria [66, 82, 94].

Thus far, the most effective malaria treatment is administration of antimalarial drugs, Chloroquine (CQ) or Artemisinin (ART) and its derivatives, which solely targets the parasite. But the emergence of CQ/ART-resistance parasite species rendered these treatments increasingly ineffective [237, 238]. In the recent years, increased knowledge of the host immune response uncovers a potential to employ host-directed therapy in malaria infection. In fact, immunotherapy has emerged as a hot topic for both research and treatment against a diverse array of disease over the last few centuries [239–241]. Specifically, interferon therapy has been widely reported to treat cancer [242–246] and viral infections [247–249]. Recently, a synthetic innate defense regulator-1018 (IDR-1018) adjunctive treatment, in combination with antimalarial drug, demonstrated efficacy against ECM [250]. Taken together, these data offer the possibility of interferon treatment as an immunotherapy for malaria infection. Thus, dissecting the innate signaling pathways and their corresponding cytokine responses would provide further insights into the induction of adaptive immune response and offer some directions on vaccine or drug developments.

## Figures and Tables

**Table 1:** Combinations of different mouse backgrounds and parasite strains combinations allow the study of many disease profiles.

Mouse strain	Infection	Infection/pathology/protection	Ref.
C57BL/6 C57BL/6J C57BL/6N C57BL/6AnNCr 129/Ola x C57BL/6 129P2Sv/Ev 129 Sv/Ev x C57BL/6	*P. berghei *ANKA	ECM	[61–76]
	*P. berghei *ANKA-luc (231c11)		[21, 77]
	*P. berghei *ANKA-GFP		[78, 79]
	*P. berghei *ANKA clone 15cy1		[80, 81]
	*P. berghei *ANKA-GFP clone 15cy1		[82]
	*P. berghei *ANKA clone BdS		[26, 83]
	*P. berghei *ANKA sporozoite		[73]
	*P. berghei *ANKA clone 15cy1 sporozoite		[80, 84]

C57BL/6	*P. berghei *K173	Protection from ECM	[67]
	*P. yoelii nigeriensis *N67C	Lethal hyperparasitemia and severe anemia	[85]
	*P. yoelii *17XNL	Protection from lethal hyperparasitemia and severe anemia	[86]
	*P. yoelii yoelii *265 BY uncloned line		[87]
	*P. yoelii nigeriensis *N67		[85]
	*P. berghei *NK65	Liver injury	[88]
		Malaria-associated acute respiratory distress syndrome	[76]
	*P. berghei *NK65-GFP	Placental malaria	[89]
	*P. chabaudi chabaudi *AS	Protection in uncomplicated malaria	[77, 90]
	*P. chabaudi *AS		[76, 91]
	*P. berghei *ANKA-luc sporozoite	Liver-stage malaria	[92]
	*P. berghei *NK65 sporozoite		
	*P. yoelii *17XNL sporozoite		

C57BL/6.C-*H2 * ^*d*^/bBy	*P. berghei *ANKA-GFP sporozoite	Liver-stage malaria	[93]

CBA/J	*P. berghei *ANKA	ECM	[75]
	*P. yoelii *17X	Protection from lethal hyperparasitemia and severe anemia	[94]
	*P. yoelii *17XNL		

CBA/T6	*P. berghei *ANKA	ECM	[95]
	*P. berghei *K173	Protection from ECM	

CBA/CaH	*P. chabaudi adami *556 KA	Protection against blood-stage malaria	[96]

DBA/2	*P. berghei *ANKA	Protection from ECM	[84]
		Acute lung injury	

129 Sv/Ev	*P. berghei *ANKA clone 1.4	ECM	[97]
	*P. chabaudi chabaudi *AS	Protection against blood-stage malaria	[98]

BALB/c	*P. berghei *ANKA	Protection from ECM	[65, 70, 74, 75, 84]
	*P. berghei *ANKA clone BdS		[26]
	*P. berghei *ANKA-GFP		[79]

BALB/cByJ SW	*P. yoelii *17XL	Lethal hyperparasitemia and severe anemia	[94]
	*P. yoelii *17XNL	Protection from lethal hyperparasitemia and severe anemia	

**Table 2:** List of malarial ligands that stimulate different signaling molecules to trigger diverse immune responses and affect disease outcome in various experimental models.

Ligand	Signalling molecules involved	Cell types/mice	Immune responses/functions	Ref.
GPI	TLR1-TLR2 heterodimer	BMDM, PBMC	Stimulates production of TNF-*α*, IL-12, IL-6, and NO	[99]
	TLR2/TLR1, TLR4, MyD88, ERK1/2, p38, JNK1/2, NF-*κ*B, AP-1 (c-Jun, ATF-2)	BMDM, PBMC, HEK, MPM		[62, 100, 101]
	MAPK2	BMDM	Stimulates production of TNF-*α* Controls production of IL-12	[102]
	I*κ*B-*ζ*	BMDM	Involved in IL-12 expression	[103]

Hz (*Pf* 3D7)/ synthetic Hz	TLR9, MyD88	Murine splenocytes, BMDDC	Stimulates production of TNF-*α*, IL-12p40, MCP-1, and IL-6	[104]
		Knockout C57BL/6 or 129/Ola x C57BL/6	Increases serum level of MCP-1 and IL-6	
	TLR2, TLR9, MyD88	Knockout C57BL/6	Involved in ECM development Promotes parasite and leukocyte sequestration in brain sections Stimulates production of IFN-*γ*, TNF-*α*, and IL-12p40	[63]

Malarial CpG DNA (*Pf* 3D7)	TLR9, MyD88	BMDDC	Stimulates production of IL-12p40 and Rantes	[105]

Host fibrinogen	TLR4, CD11b/CD18-integrin	PBMC	Stimulates release of ROS, TNF, and MCP-1	[106]

Heme	TLR4, CD14, MyD88, I*κ*B-*α*, ERK1/2, NF-*κ*B	MPM, BMDDC, human monocyte-derived macrophages, PBMC	Stimulates production of TNF-*α* and KC Controls release of PGE_2_	[107, 108]

MPs from infected mouse	TLR4, MyD88	BMDM	Upregulate expression of CD40 Stimulate production of TNF	[109]

Malarial AT-rich motif	STING, TBK1, IRF-3, IRF-7	BMDM, HEK293, knockout C57BL/6	Involved in ECM development Stimulates production of IFN-I, TNF-*α*, IL-6, and IL-15	[61]

*Plasmodium* DNA/RNA	MDA5, MAVS, RIG-1, CD14/IL-1R, p38	Knockout C57BL/6, RAW264.7	Stimulates production of IFN-I Controls parasitemia Prevents parasite sequestration in the brain capillaries and apoptosis in the spleen Promotes phagocytosis activity of macrophages	[85]

Unknown in PbA infection	TLR2/4, MyD88	Knockout C57BL/6	Involved in ECM development initiated with sporozoites Partially involved in ECM development initiated with iRBCs Regulates production of IFN-*γ*, MCP-1, TNF-*α*, and IL-10	[80]

Unknown ligand in *Py* 17XNL infection	TLR9, MyD88	Knockout C57BL/6	Controls parasitemia and promotes survival Essential Th1 development and cell-mediated immunity Stimulates production of TNF-*α* and IL-12 by DC Controls production of IL-10 and IL-4 by DC Induces cytotoxic activity in NK and CD8^+^ T cells	[86]

Unknown	TLR7, TLR9	NK cells, *γδ* T cells, CD4^+^ T cells	TLR7 mediates IFN-*γ* production by NK cells 24 h after infection TLR7 stimulates production of IFN-I, IFN-*γ*, IL-10, and IL-12 TLR9 mediates IFN-*γ* production by CD4^+^ cells and stimulates production of TNF 6 days after infection	[110]

*Plasmodium* RNA	MDA5, MAVS, IRF-3, IRF-7	Knockout C57BL/6J, BMDDC, MPH	Stimulates production of IFN-I Controls leukocyte recruitment which limits parasite growth in the liver and induction of erythrocytic stage infection	[92]

*Pf*TyrRs	Unknown	Mouse splenocytes, PBMC, RAW 264.7, THP-1	Stimulates production of TNF-*α*, IL-6, IL-1*α*, and IL-1*β* which upregulate expression of ICAM-1 and VCAM-1 receptors	[111]

*Pf*HMGB	Unknown	Mouse splenocytes, RAW 264.7	Stimulates production of TNF-*α*, IL-6, IL-8, and IL-1*β* and upregulates mRNA expression of iNOS	[112]

BMDM: mouse bone marrow-derived macrophages (C57BL/6 unless otherwise stated); BMDDC: mouse bone marrow-derived dendritic cells; PBMC: human peripheral blood mononuclear cells; HEK: human embryonic kidney epithelial cells; Hz: Hemozoin; iNOS: inducible nitric oxide synthetase; KC: keratinocyte chemokine; MCP-1: monocyte chemoattractant protein-1; MP: microparticles; MPH: mouse primary hepatocytes; MPM: murine peritoneal macrophages; PGE_2_: prostaglandin E2; *Pf*TyrRS: *P. falciparum *tyrosyl-tRNA synthetase; *Pf*HMGB: *P. falciparum *high mobility group box protein; RAW264.7: murine macrophage-like cell line; ROS: reactive oxide species; THP-1: human monocytic leukemia cell line.

**Table 3:** Association of TLRs and adaptor molecules gene polymorphisms with susceptibility to malaria or pathology in human.

TLRs/ adaptors	SNPs	Association	Region	Ref.
TLR1	S248N rs4833095	Placental malaria and anemia	Ghana	[113]
	I602S	Susceptibility to malaria infection	Amazon	[114]

TLR2	Δ22	No association with serum cytokines (TNF, IFN-*γ*, IL-1*β*, IL-6, IL-10) levels	Uganda	[115]
		Susceptibility to cerebral malaria		
	GT_*n*_	No association with serum cytokines (TNF, IFN-*γ*, IL-1*β*, IL-6, IL-10) levels		
		No association with cerebral malaria		

TLR4	D299G rs4986790	No association with susceptibility to malaria infection	Burundi, Amazon, Ghana, Iran	[114, 116–118]
		No association with risk of placental malaria	Ghana	[118]
		Maternal anemia		
		Severe malaria		[119]
	T399I	No association with mild malaria	Iran	[116]
		Severe malaria	Ghana	[119]

TLR6	S249P	Susceptibility to mild malaria	Amazon	[114]

TLR9	G1174A rs352139	No association with susceptibility to malaria infection	Burundi	[117]
		Susceptibility to mild malaria	Ghana	[120]
		Level of parasitemia		
		No association with serum TNF*α* level	Uganda	[121]
		No association with serum IFN-*γ* level in mild malaria children		
		Level of serum IFN-*γ* level in CM children		
	T1237C rs5743836	No association with susceptibility to malaria infection	Ghana, Iran	[116, 118, 120]
		No association with disease severity	Ghana	[119]
		No association with placental malaria		[118]
		Susceptibility to malaria infection	Burundi	[117]
		Level of parasitemia	Amazon, Ghana	[114, 120]
		No association with serum TNF*α* level	Uganda	[121]
		No association with serum IFN-*γ* level in mild malaria children		
		Level of serum IFN-*γ* level in CM children		
	T1486C rs187084	No association with susceptibility to malaria infection	Burundi, Ghana, Iran	[116–118, 120]
		No association with disease severity	Ghana	[119]
		No association with placental malaria		[118]
		No association with level of parasitemia		[120]
		Level of parasitemia	Amazon	[114]
	G2848A rs352140	No association with level of parasitemia	Ghana	[120]
		Susceptibility to mild malaria infection		

TIRAP	S180L rs8177374	No association with susceptibility to malaria or severity of infection	Burundi, Amazon	[114, 117]
		Mild malaria and severe malaria	Gambia, Vietnam, Kenya	[122]
		Mild malaria	Iran	[116]

CM: cerebral malaria; Δ22: 22 base pair deletion in the first untranslated exon; GT_*n*_: GT dinucleotide repeat in the second intron; mild malaria: patients suffer fever with temperature greater than or equal to 38°C, malaise, muscular pain, headache, and parasite load greater than or equal to 5000 parasite/ul of blood; severe malaria: patients who suffer anaemia, prostration, respiratory distress, convulsions, and/or impaired consciousness; cerebral malaria (CM): patients who experience coma with *P. falciparum *on blood smear and have no other cause for coma.

**Table 4:** Diverse roles of different IRFs in malaria infection.

	Host /model	Infection	Functions	Ref.
IRF-1	Human	*P. falciparum *	Controls parasitemia	[123]
			Not involved in development of severe malaria	[124]
	Mice	*Py nigeriensis *N67 iRBCs	Plays a role in IFN-I signaling	[85]
		PbA iRBCs	Involved in ECM development Controls parasitemia	[69]
			Involved in ECM development Promotes parasitemia	[125]
			Regulates production of IFN-*γ* and IL12p4 Controls CD8^+^ T cells numbers	[72]
			Plays a role in ECM development	[70, 95]
	*Ex vivo *	AT-rich oligonucleotides	No effect on IFN-*β* production by splenocytes	[61]

IRF-3	Mice	*P. yoelii *sporozoite	Mediates IFN-I-induced innate response during liver-stage infection	[126]
		*P. chabaudi *iRBCs	Mediates splenic IFN-*α*, but not IFN-*β*, transcription in red pulp macrophages	[90]
			Not involved in IFN-I production	[110]

IRF-5	*Ex vivo *	AT-rich oligonucleotides	Not involved in IFN-*β* production by splenocytes	[61]

IRF-7	Mice	*Py nigeriensis *N67 iRBCs	Plays a role in IFN-I signaling	[85]
		PbA iRBCs	Plays a role in ECM development	[70, 71]
		*P.chabaudi *iRBCs	Mediates splenic IFN-I transcription in red pulp macrophages	[90]
			Involved in IFN-I production	[110]

IRF-3 and IRF-7	Mice	PbA sporozoite	Mediate IFN-I response in liver-stage infection Control parasite load in the liver	[92]
		PbA iRBCs	Involved in ECM development	[61]
	*Ex vivo *	AT-rich oligonucleotides	Mediate IFN-*β* production by splenocytes	
		*Pf *iRBCs	Mediate IFN-*β* production by macrophages	

IRF-8	Mice	PbA iRBCs	Plays a role in ECM development	[95]
			Regulates production of proinflammatory cytokines Mediates IFN-I production Controls antigen processing and presentation and chemotaxis	[72]

IRF-9	Mice	*Py nigeriensis *N67 iRBCs	Plays a role in IFN-I signaling	[85]
		PbA iRBCs	Plays a role in ECM development	[72]

## References

[1] WHO (2013). World malaria report 2013 shows major progress in fight against malaria, calls for sustained financing.

[2] Cowman AF, Kappe SHI (2006). Malaria's stealth shuttle. *Science*.

[3] Giboda M, Gutvirth J, Maloveská M, Kosina F, Hocmanová M, Struncová V (1987). Imported malaria diagnostic and clinical features. *Bratislavske Lekarske Listy*.

[4] Perkins DJ, Were T, Davenport GC, Kempaiah P, Hittner JB, Ong'echa JM (2011). Severe malarial anemia: innate immunity and pathogenesis. *International Journal of Biological Sciences*.

[5] Taylor WRJ, Hanson J, Turner GDH, White NJ, Dondorp AM (2012). Respiratory manifestations of malaria. *Chest*.

[6] Idro R, Jenkins NE, Newton CRJ (2005). Pathogenesis, clinical features, and neurological outcome of cerebral malaria. *The Lancet Neurology*.

[7] Rénia L, Marussig MS, Grillot D (1991). *In vitro* activity of CD4^+^ and CD8^+^ T lymphocytes from mice immunized with a synthetic malaria peptide. *Proceedings of the National Academy of Sciences of the United States of America*.

[8] Schofield L, Grau GE (2005). Immunological processes in malaria pathogenesis. *Nature Reviews Immunology*.

[9] Horne-Debets JM, Faleiro R, Karunarathne DS (2013). PD-1 dependent exhaustion of CD8+ T cells drives chronic malaria. *Cell Reports*.

[10] Ewer KJ, O’Hara GA, Duncan CJA (2013). Protective CD8^+^ T-cell immunity to human malaria induced by chimpanzee adenovirus-MVA immunisation. *Nature Communications*.

[11] Artavanis-Tsakonas K, Riley EM (2002). Innate immune response to malaria: rapid induction of IFN-*γ* from human NK cells by live Plasmodium falciparum-infected erythrocytes. *The Journal of Immunology*.

[12] Santhanam J, Råberg L, Jon Savill N (2014). Immune-mediated competition in rodent Malaria is most likely caused by induced changes in innate immune clearance of merozoites. *PLoS Computional Biology*.

[13] Molineaux L, Träuble M, Collins WE, Jeffery GM, Dietz K (2002). Malaria therapy reinculation data suggest individual variation of an innate immune response and independent acquisition of antiparasitic and antitoxic immunities. *Transactions of the Royal Society of Tropical Medicine and Hygiene*.

[14] Scragg IG (1999). Early cytokine induction by *Plasmodium falciparum* is not a classical endotoxin-like process. *European Journal of Immunology*.

[15] Brown H, Turner G, Rogerson S (1999). Cytokine expression in the brain in human cerebral malaria. *Journal of Infectious Diseases*.

[16] Angulo I, Fresno M (2002). Cytokines in the pathogenesis of and protection against malaria. *Clinical and Diagnostic Laboratory Immunology*.

[17] Tjitra E, Anstey NM, Sugiarto P (2008). Multidrug-resistant Plasmodium vivax associated with severe and fatal malaria: a prospective study in Papua, Indonesia. *PLoS Medicine*.

[18] Ahamada S, Wery M, Hamers R (1994). Rodent malaria parasites: molecular karyotypes characterize species, subspecies and lines. *Parasite*.

[19] Perkins SL, Sarkar IN, Carter R (2007). The phylogeny of rodent malaria parasites: simultaneous analysis across three genomes. *Infection, Genetics and Evolution*.

[20] Amante FH, Haque A, Stanley AC (2010). Immune-mediated mechanisms of parasite tissue sequestration during experimental cerebral malaria. *Journal of Immunology*.

[21] Claser C, Malleret B, Gun SY (2011). Cd8^+^ T cells and IFN-*γ* mediate the time-dependent accumulation of infected red blood cells in deep organs during experimental cerebral malaria. *PLoS ONE*.

[22] McQuillan JA, Mitchell AJ, Ho YF (2011). Coincident parasite and CD8 T cell sequestration is required for development of experimental cerebral malaria. *International Journal for Parasitology*.

[23] El-Assaad F, Whewaya J, Mitchell AJ, et al (2013). Cytoadherence of *Plasmodium* berghei-infected red blood cells to murine brain and lung microvascular endothelial cells *in vitro*. *Infection and Immunity*.

[24] Newton CRJC, Hien TT, White N (2000). Cerebral malaria. *Journal of Neurology Neurosurgery & Psychiatry*.

[25] Wassmer SC, Combes V, Grau GE (2003). Pathophysiology of cerebral malaria: Role of host cells in the modulation of cytoadhesion. *Annals of the New York Academy of Sciences*.

[26] Belnoue E, Kayibanda M, Vigario AM (2002). On the pathogenic role of brain-sequestered alphabeta CD8+ T cells in experimental cerebral malaria. *Journal of Immunology*.

[27] Howland SW, Poh CM, Gun SY (2013). Brain microvessel cross-presentation is a hallmark of experimental cerebral malaria. *EMBO Molecular Medicine*.

[28] Medzhitov R, Janeway C. J (2000). Advances in immunology: innate immunity. *The New England Journal of Medicine*.

[29] Medzhitov R, Janeway C (2000). Innate immune recognition: Mechanisms and pathways. *Immunological Reviews*.

[30] Akira S, Uematsu S, Takeuchi O (2006). Pathogen recognition and innate immunity. *Cell*.

[31] Vasselon T, Detmers PA (2002). Toll receptors: a central element in innate immune responses. *Infection and Immunity*.

[32] Kawai T, Akira S (2008). Toll-like receptor and RIG-1-like receptor signaling. *Annals of the New York Academy of Sciences*.

[33] Chtarbanova S, Imler J (2011). Microbial sensing by toll receptors: a historical perspective. *Arteriosclerosis, Thrombosis, and Vascular Biology*.

[34] Cambi A, Figdor CG (2003). Dual function of C-type lectin-like receptors in the immune system. *Current Opinion in Cell Biology*.

[35] Pyz E, Marshall ASJ, Gordon S, Brown GD (2006). C-type lectin-like receptors on myeloid cells. *Annals of Medicine*.

[36] Geijtenbeek TBH, Gringhuis SI (2009). Signalling through C-type lectin receptors: shaping immune responses. *Nature Reviews Immunology*.

[37] Kanneganti T-D, Lamkanfi M, Núñez G (2007). Intracellular NOD-like receptors in host defense and disease. *Immunity*.

[38] Proell M, Riedl SJ, Fritz JH, Rojas AM, Schwarzenbacher R (2008). The Nod-Like Receptor (NLR) family: a tale of similarities and differences. *PLoS ONE*.

[39] Geddes K, Magalhães JG, Girardin SE (2009). Unleashing the therapeutic potential of NOD-like receptors. *Nature Reviews Drug Discovery*.

[40] Loo YM, Gale M (2011). Immune Signaling by RIG-I-like Receptors. *Immunity*.

[41] Muzio M, Bosisio D, Polentarutti N (2000). Differential expression and regulation of toll-like receptors (TLR) in human leukocytes: selective expression of TLR3 in dendritic cells. *Journal of Immunology*.

[42] Hornung V, Rothenfusser S, Britsch S (2002). Quantitative expression of toll-like receptor 1-10 mRNA in cellular subsets of human peripheral blood mononuclear cells and sensitivity to CpG oligodeoxynucleotides. *Journal of Immunology*.

[43] Jarrossay D, Napolitani G, Colonna M, Sallusto F, Lanzavecchia A (2001). Specialization and complementarity in microbial molecule recognition by human myeloid and plasmacytoid dendritic cells. *European Journal of Immunology*.

[44] Ito T, Amakawa R, Kaisho T (2002). Interferon-*α* and interleukin-12 are induced differentially by toll-like receptor 7 ligands in human blood dendritic cell subsets. *Journal of Experimental Medicine*.

[45] Geijtenbeek TBH, Torensma R, Van Vliet SJ (2000). Identification of DC-SIGN, a novel dendritic cell-specific ICAM-3 receptor that supports primary immune responses. *Cell*.

[46] Poulin LF, Reyal Y, Uronen-Hansson H (2012). DNGR-1 is a specific and universal marker of mouse and human Batf3-dependent dendritic cells in lymphoid and nonlymphoid tissues. *Blood*.

[47] Smits HH, Engering A, Van Der Kleij D (2005). Selective probiotic bacteria induce IL-10-producing regulatory T cells in vitro by modulating dendritic cell function through dendritic cell-specific intercellular adhesion molecule 3-grabbing nonintegrin. *Journal of Allergy and Clinical Immunology*.

[48] Geijtenbeek TB, van Vliet SJ, Koppel EA (2003). Mycobacteria target DC-SIGN to suppress dendritic cell function. *Journal of Experimental Medicine*.

[49] Bergman MP, Engering A, Smits HH (2004). Helicobacter pylori modulates the T helper cell 1/T helper cell 2 balance through phase-variable interaction between lipopolysaccharide and DC-SIGN. *Journal of Experimental Medicine*.

[50] Li J, Ma Z, Tang Z, Stevens T, Pitt B, Li S (2004). CpG DNA-mediated immune response in pulmonary endothelial cells. *The American Journal of Physiology—Lung Cellular and Molecular Physiology*.

[51] Guillott L, Medjane S, Le-Barillec K (2004). Response of human pulmonary epithelial cells to lipopolysaccharide involves toll-like receptor 4 (TLR4)-dependent signaling pathways: Evidence for an intracellular compartmentalization of TLR4. *The Journal of Biological Chemistry*.

[52] Droemann D, Goldmann T, Branscheid D (2003). Toll-like receptor 2 is expressed by alveolar epithelial cells type II and macrophages in the human lung. *Histochemistry and Cell Biology*.

[53] Claeys S, de Belder T, Holtappels G (2003). Human *β*-defensins and toll-like receptors in the upper airway. *Allergy*.

[54] Maaser C, Heidemann J, Von Eiff C (2004). Human intestinal microvascular endothelial cells express Toll-like receptor 5: a binding partner for bacterial flagellin. *Journal of Immunology*.

[55] Cario E, Podolsky DK (2000). Differential alteration in intestinal epithelial cell expression of Toll-like receptor 3 (TLR3) and TLR4 in inflammatory bowel disease. *Infection and Immunity*.

[56] Gewirtz AT, Navas TA, Lyons S, Godowski PJ, Madara JL (2001). Cutting edge: bacterial flagellin activates basolaterally expressed TLR5 to induce epithelial proinflammatory gene expression. *Journal of Immunology*.

[57] Frantz S, Kobzik L, Kim Y (1999). Toll4 (TLR4) expression in cardiac myocytes in normal and failing myocardium. *Journal of Clinical Investigation*.

[58] Bulut Y, Faure E, Thomas L (2002). Chlamydial heat shock protein 60 activates macrophages and endothelial cells through toll-like receptor 4 and MD2 in a MyD88-dependent pathway. *The Journal of Immunology*.

[59] Imaizumi T, Aratani S, Nakajima T (2002). Retinoic acid-inducible gene-I is induced in endothelial cells by LPS and regulates expression of COX-2. *Biochemical and Biophysical Research Communications*.

[60] Opitz B, Förster S, Hocke AC (2005). Nod1-mediated endothelial cell activation by Chlamydophila pneumoniae. *Circulation Research*.

[61] Sharma S, DeOliveira RB, Kalantari P (2011). Innate immune recognition of an AT-rich stem-loop DNA motif in the Plasmodium falciparum genome. *Immunity*.

[62] Lu Z, Serghides L, Patel SN (2006). Disruption of JNK2 decreases the cytokine response to *Plasmodium falciparum* glycosylphosphatidylinositol in vitro and confers protection in a cerebral malaria model. *Journal of Immunology*.

[63] Coban C, Uematsu S, Arisue N (2007). Pathological role of Toll-like receptor signaling in cerebral malaria. *International Immunology*.

[64] Lepenies B, Cramer JP, Burchard GD, Wagner H, Kirschning CJ, Jacobs T (2008). Induction of experimental cerebral malaria is independent of TLR2/4/9. *Medical Microbiology and Immunology*.

[65] Griffith JW, O'Connor C, Bernard K, Town T, Goldstein DR, Bucala R (2007). Toll-like receptor modulation of murine cerebral malaria is dependent on the genetic background of the host. *Journal of Infectious Diseases*.

[66] Rudin W, Favre N, Bordmann G, Ryffel B (1997). Interferon-*γ* is essential for the development of cerebral malaria. *European Journal of Immunology*.

[67] Mitchell AJ, Hansen AM, Hee L (2005). Early cytokine production is associated with protection from murine cerebral malaria. *Infection and Immunity*.

[68] Morrell CN, Srivastava K, Swaim A (2011). Beta interferon suppresses the development of experimental cerebral malaria. *Infection and Immunity*.

[69] Senaldi G, Shaklee CL, Guo J (1999). Protection against the mortality associated with disease models mediated by TNF and IFN-*γ* in mice lacking IFN regulatory factor-1. *Journal of Immunology*.

[70] Lovegrove FE, Gharib SA, Patel SN, Hawkes CA, Kain KC, Liles WC (2007). Expression microarray analysis implicates apoptosis and interferon-responsive mechanisms in susceptibility to experimental cerebral malaria. *The American Journal of Pathology*.

[71] Sexton AC, Good RT, Hansen DS (2004). Transcriptional profiling reveals suppressed erythropoiesis, up-regulated glycolysis, and interferon-associated responses in murine malaria. *Journal of Infectious Diseases*.

[72] Berghout J, Langlais D, Gros P (2013). Irf8-regulated genomic responses drive pathological inflammation during cerebral Malaria. *PLoS Pathogens*.

[73] Hafalla JCR, Burgold J, Dorhoi A (2012). Experimental cerebral malaria develops independently of caspase recruitment domain-containing protein 9 signaling. *Infection and Immunity*.

[74] Lovegrove FE, Peña-Castillo L, Mohammad N, Liles WC, Hughes TR, Kain KC (2006). Simultaneous host and parasite expression profiling identifies tissue-specific transcriptional programs associated with susceptibility or resistance to experimental cerebral malaria. *BMC Genomics*.

[75] Delahaye NF, Coltel N, Puthier D (2007). Gene expression analysis reveals early changes in several molecular pathways in cerebral malaria-susceptible mice versus cerebral malaria-resistant mice. *BMC Genomics*.

[76] van den Steen PE, Geurts N, Deroost K (2010). Immunopathology and dexamethasone therapy in a new model for malaria-associated acute respiratory distress syndrome. *American Journal of Respiratory and Critical Care Medicine*.

[77] Haque A, Best SE, Ammerdorffer A (2011). Type I interferons suppress CD4 + T-cell-dependent parasite control during blood-stage Plasmodium infection. *European Journal of Immunology*.

[78] Togbe D, Schofield L, Grau GE (2007). Murine cerebral malaria development is independent of toll-like receptor signaling. *The American Journal of Pathology*.

[79] Ball EA, Sambo MR, Martins M (2013). IFNAR1 controls progression to cerebral malaria in children and CD8 + T cell brain pathology in plasmodium berghei-infected mice. *Journal of Immunology*.

[80] Kordes M, Matuschewski K, Hafalla JCR (2011). Caspase-1 activation of interleukin-1ß (IL-1ß) and IL-18 Is dispensable for induction of experimental cerebral malaria. *Infection and Immunity*.

[81] Villegas-Mendez A, Greig R, Shaw TN (2012). IFN-*γ*-producing CD4^+^ T cells promote experimental cerebral malaria by modulating CD8^+^ T cell accumulation within the brain. *The Journal of Immunology*.

[82] Palomo J, Fauconnier M, Coquard L (2013). Type I interferons contribute to experimental cerebral malaria development in response to sporozoite or blood-stage *Plasmodium berghei* ANKA. *European Journal of Immunology*.

[83] Belnoue E, Potter SM, Rosa DS (2008). Control of pathogenic CD8^+^ T cell migration to the brain by IFN-*γ* during experimental cerebral malaria. *Parasite Immunology*.

[84] Epiphanio S, Campos MG, Pamplona A (2010). VEGF promotes malaria-associated acute lung injury in mice. *PLoS Pathogens*.

[85] Wu J, Tian L, Yu X (2014). Strain-specific innate immune signaling pathways determine malaria parasitemia dynamics and host mortality. *Proceedings of the National Academy of Sciences*.

[86] Gowda NM, Wu X, Gowda DC (2012). TLR9 and MyD88 are crucial for the development of protective immunity to malaria. *The Journal of Immunology*.

[87] Vigário AM, Belnoue E, Cumano A (2001). Inhibition of Plasmodium yoelii blood-stage malaria by interferon *α* through the inhibition of the production of its target cell, the reticulocyte. *Blood*.

[88] Adachi K, Tsutsui H, Kashiwamura SI (2001). Plasmodium berghei infection in mice induces liver injury by an IL-12- and toll-like receptor/myeloid differentiation factor 88-dependent mechanism. *Journal of Immunology*.

[89] Barboza R, Reis AS, da Silva LG (2013). MyD88 signaling is directly involved in the development of murine placental malaria. *Infection and Immunity*.

[90] Kim CC, Nelson CS, Wilson EB, Hou B, DeFranco AL, DeRisi JL (2012). Splenic red pulp macrophages produce type I interferons as early sentinels of malaria infection but are dispensable for control. *PLoS ONE*.

[91] Su Z, Stevenson MM (2000). Central role of endogenous gamma interferon in protective immunity against blood-stage Plasmodium chabaudi AS infection. *Infection and Immunity*.

[92] Liehl P, Zuzarte-Luís V, Chan J (2014). Host-cell sensors for Plasmodium activate innate immunity against liver-stage infection. *Nature Medicine*.

[93] Gonçalves LA, Rodrigues-Duarte L, Rodo J, Vieira de Moraes L, Marques I, Penha-Gonçalves C (2013). TREM2 governs Kupffer cell activation and explains belr1 genetic resistance to malaria liver stage infection. *Proceedings of the National Academy of Sciences of the United States of America*.

[94] Shear HL, Srinivasan R, Nolan T, Ng C (1989). Role of IFN-*γ* in lethal and nonlethal malaria in susceptible and resistant murine hosts. *Journal of Immunology*.

[95] Miu J, Hunt NH, Ball HJ (2008). Predominance of interferon-related responses in the brain during murine malaria, as identified by microarray analysis. *Infection and Immunity*.

[96] Clark IA, Hunt NH, Butcher GA, Cowden WB (1987). Inhibition of murine malaria (Plasmodium chabaudi) in vivo by recombinant interferon-*γ* or tumor necrosis factor, and its enhancement by butylated hydroxyanisole. *The Journal of Immunology*.

[97] Amani V, Vigário AM, Belnoue E (2000). Involvement of IFN-*γ* receptor-mediated signaling in pathology and anti-malarial immunity induced by Plasmodium berghei infection. *European Journal of Immunology*.

[98] Voisine C, Mastelic B, Sponaas A, Langhorne J (2010). Classical CD11c^+^ dendritic cells, not plasmacytoid dendritic cells, induce T cell responses to *Plasmodium chabaudi* malaria. *International Journal for Parasitology*.

[99] Zhu J, Krishnegowda G, Li G, Channe Gowda D (2011). Proinflammatory responses by glycosylphosphatidylinositols (GPIs) of Plasmodium falciparum are mainly mediated through the recognition of TLR2/TLR1. *Experimental Parasitology*.

[100] Zhu J, Krishnegowda G, Gowda DC (2005). Induction of proinflammatory responses in macrophages by the glycosylphosphatidylinositols of Plasmodium falciparum: The requirement of extracellular signal-regulated kinase, p38, c-Jun N-terminal kinase and NF-*κ*B pathways for the expression of proinflammatory cytokines and nitric oxide. *Journal of Biological Chemistry*.

[101] Krishnegowda G, Hajjar AM, Zhu J (2005). Induction of proinflammatory responses in macrophages by the glycosylphosphatidylinositols of Plasmodium falciparum: cell signaling receptors, glycosylphosphatidylinositol (GPI) structural requirement, and regulation of GPI activity. *Journal of Biological Chemistry*.

[102] Zhu J, Wu X, Goel S (2009). MAPK-activated protein kinase 2 differentially regulates plasmodium falciparum glycosylphosphatidylinositol-inducedproduction of tumor necrosis Factor-*α* and interleukin-12 in macrophages. *The Journal of Biological Chemistry*.

[103] Zhu J, Weinberg R, Wu X, Gowda NM, Muta T, Gowda DC (2012). Ikappab-zeta plays an important role in the ERK-dependent dysregulation of malaria parasite GPI-induced IL-12 expression. *IUBMB Life*.

[104] Coban C, Ishii KJ, Kawai T (2005). Toll-like receptor 9 mediates innate immune activation by the malaria pigment hemozoin. *The Journal of Experimental Medicine*.

[105] Parroche P, Lauw FN, Goutagny N (2007). Malaria hemozoin is immunologically inert but radically enhances innate responses by presenting malaria DNA to Toll-like receptor 9. *Proceedings of the National Academy of Sciences of the United States of America*.

[106] Barrera V, Skorokhod OA, Baci D, Gremo G, Arese P, Schwarzer E (2011). Host fibrinogen stably bound to hemozoin rapidly activates monocytes via TLR-4 and CD11b/CD18-integrin: A new paradigm of hemozoin action. *Blood*.

[107] Andrade BB, Araújo-Santos T, Luz NF (2010). Heme impairs prostaglandin E2 and TGF-*β* production by human mononuclear cells via Cu/Zn superoxide dismutase: insight into the pathogenesis of severe malaria. *The Journal of Immunology*.

[108] Figueiredo RT, Fernandez PL, Mourao-Sa DS (2007). Characterization of heme as activator of toll-like receptor 4. *The Journal of Biological Chemistry*.

[109] Couper KN, Barnes T, Hafalla JCR (2010). Parasite-derived plasma microparticles contribute significantly to malaria infection-induced inflammation through potent macrophage stimulation. *PLoS Pathogens*.

[110] Baccarella A, Fontana MF, Chen E, Kim CC (2013). Toll-like receptor 7 mediates early innate immune responses to malaria. * Infection and Immunity*.

[111] Bhatt TK, Sharma A, Khan S (2011). Malaria parasite tyrosyl-tRNA synthetase secretion triggers pro-inflammatory responses. *Nature Communications*.

[112] Kumar K, Singal A, Rizvi MMA, Chauhan VS (2008). High mobility group box (HMGB) proteins of Plasmodium falciparum: DNA binding proteins with pro-inflammatory activity. *Parasitology International*.

[113] Hamann L, Bedu-Addo G, Eggelte TA, Schumann RR, Mockenhaupt FP (2010). The toll-like receptor 1 variant S248N influences placental malaria. *Infection, Genetics and Evolution*.

[114] Leoratti FMS, Farias L, Alves FP (2008). Variants in the toll-like receptor signaling pathway and clinical outcomes of malaria. *The Journal of Infectious Diseases*.

[115] Greene JA, Sam-Agudu N, John CC, Opoka RO, Zimmerman PA, Kazura JW (2012). Toll-like receptor polymorphisms and cerebral malaria: TLR2 Δ22 polymorphism is associated with protection from cerebral malaria in a case control study. *Malaria Journal*.

[116] Zakeri S, Pirahmadi S, Mehrizi AA, Djadid ND (2011). Genetic variation of *TLR-4*, *TLR-9* and *TIRAP* genes in Iranian malaria patients. *Malaria Journal*.

[117] Esposito S, Molteni CG, Zampiero A (2012). Role of polymorphisms of toll-like receptor (TLR) 4, TLR9, toll-interleukin 1 receptor domain containing adaptor protein (TIRAP) and FCGR2A genes in malaria susceptibility and severity in Burundian children. *Malaria Journal*.

[118] Mockenhaupt FP, Hamann L, Von Gaertner C (2006). Common polymorphisms of toll-like receptors 4 and 9 are associated with the clinical manifestation of malaria during pregnancy. *Journal of Infectious Diseases*.

[119] Mockenhaupt FP, Cramer JP, Hamann L (2006). Toll-like receptor (TLR) polymorphisms in African children: common TLR-4 variants predispose to severe malaria. *Proceedings of the National Academy of Sciences of the United States of America*.

[120] Omar AH, Yasunami M, Yamazaki A (2012). Toll-like receptor 9 (TLR9) polymorphism associated with symptomatic malaria: a cohort study. *Malaria Journal*.

[121] Sam-Agudu NA, Greene JA, Opoka RO (2010). TLR9 polymorphisms are associated with altered IFN-*γ*levels in children with cerebral malaria. *The American Journal of Tropical Medicine and Hygiene*.

[122] Khor CC, Chapman SJ, Vannberg FO (2007). A Mal functional variant is associated with protection against invasive pneumococcal disease, bacteremia, malaria and tuberculosis. *Nature Genetics*.

[123] Mangano VD, Luoni G, Rockett KA (2008). Interferon regulatory factor-1 polymorphisms are associated with the control of *Plasmodium falciparum* infection. *Genes & Immunity*.

[124] Mangano VD, Clark TG, Auburn S (2009). Lack of association of *interferon regulatory factor 1* with severe malaria in affected child-parental trio studies across three African populations. *PLoS ONE*.

[125] Tan RS, Feng C, Asano Y, Kara AU (1999). Altered immune response of interferon regulatory factor 1-deficient mice against Plasmodium berghei blood-stage malaria infection. *Infection and Immunity*.

[126] Miller JL, Sack BK, Baldwin M, Vaughan AM, Kappe SH (2014). Interferon-mediated innate immune responses against malaria parasite liver stages. *Cell Reports*.

[127] Gay NJ, Gangloff M (2007). Structure and function of toll receptors and their ligands. *Annual Review of Biochemistry*.

[128] Takeuchi O, Akira S (2010). Pattern recognition receptors and inflammation. *Cell*.

[129] Pietras EM, Saha SK, Cheng G (2006). The interferon response to bacterial and viral infections. *Journal of Endotoxin Research*.

[130] Perry AK, Chen G, Zheng D, Tang H, Cheng G (2005). The host type I interferon response to viral and bacterial infections. *Cell Research*.

[131] Currier J, Beck H, Currie B, Good MF (1995). Antigens released at schizont burst stimulate Plasmodium falciparum-specific CD4+ T cells from non-exposed donors: Potential for cross-reactive memory T cells to cause disease. *International Immunology*.

[132] O'Dea KP, Pasvol G (2003). Optimal tumor necrosis factor induction by Plasmodium falciparum requires the highly localized release of parasite products. *Infection and Immunity*.

[133] Kwiatkowski D, Cannon JG, Manogue KR, Cerami A, Dinarello CA, Greenwood BM (1989). Tumour necrosis factor production in Falciparum malaria and its association with schizont rupture. *Clinical and Experimental Immunology*.

[134] Kitchen SF (1949). Symptomology: general considerations. *Malariology: A Comprehensive Survey of all Aspects of this Group of Diseases from a Global Standpoint*.

[135] Gerold P, Schofield L, Blackman MJ, Holder AA, Schwarz RT (1996). Structural analysis of the glycosyl-phosphatidylinositol membrane anchor of the merozoite surface proteins-1 and -2 of *Plasmodium falciparum*. *Molecular and Biochemical Parasitology*.

[136] Goel VK, Li X, Chen H, Liu SC, Chishti AH, Oh SS (2003). Band 3 is a host receptor binding merozoite surface protein 1 during the *Plasmodium falciparum* invasion of erythrocytes. *Proceedings of the National Academy of Sciences of the United States of America*.

[137] Riglar DT, Richard D, Wilson DW (2011). Super-resolution dissection of coordinated events during malaria parasite invasion of the human erythrocyte. *Cell Host and Microbe*.

[138] Dvorak JA, Miller LH, Whitehouse WC, Shiroishi T (1975). Invasion of erythrocytes by malaria merozoites. *Science*.

[139] Boyle MJ, Langer C, Chan JA (2014). Sequential processing of merozoite surface proteins during and after erythrocyte invasion by *Plasmodium falciparum*. *Infection and Immunity*.

[140] Aikawa M, Miller LH, Johnson J, Rabbege J (1978). Erythrocyte entry by malarial parasites. A moving junction between erythrocyte and parasite. *Journal of Cell Biology*.

[141] Chugh M, Sundararaman V, Kumar S (2013). Protein complex directs hemoglobin-to-hemozoin formation in *Plasmodium falciparum*. *Proceedings of the National Academy of Sciences of the United States of America*.

[142] Kapishnikov S, Weiner A, Shimoni E (2012). Oriented nucleation of hemozoin at the digestive vacuole membrane in Plasmodium falciparum. *Proceedings of the National Academy of Sciences of the United States of America*.

[143] Kapishnikov S, Weiner A, Shimoni E, Schneider G, Elbaum M, Leiserowitz L (2013). Digestive vacuole membrane in *Plasmodium falciparum* infected erythrocytes: relevance to templated nucleation of hemozoin. *Langmuir*.

[144] Abkarian M, Massiera G, Berry L, Roques M, Braun-Breton C (2011). A novel mechanism for egress of malarial parasites from red blood cells. *Blood*.

[145] Coban C, Igari Y, Yagi M (2010). Immunogenicity of whole-parasite vaccines against *Plasmodium falciparum* involves malarial hemozoin and host TLR9. *Cell Host and Microbe*.

[146] Hashimoto C, Hudson KL, Anderson KV (1988). The Toll gene of drosophila, required for dorsal-ventral embryonic polarity, appears to encode a transmembrane protein. *Cell*.

[147] Medzhitov R, Preston-Hurlburt P, Janeway CA (1997). A human homologue of the Drosophila toll protein signals activation of adaptive immunity. *Nature*.

[148] Pandey S, Agrawal DK (2006). Immunobiology of Toll-like receptors: emerging trends. *Immunology and Cell Biology*.

[149] Chuang T, Ulevitch RJ (2001). Identification of hTLR10: a novel human Toll-like receptor preferentially expressed in immune cells. *Biochimica et Biophysica Acta: Gene Structure and Expression*.

[150] Yarovinsky F (2014). Innate immunity to Toxoplasma gondii infection. *Nature Reviews Immunology*.

[151] Kawai T, Akira S (2005). Pathogen recognition with Toll-like receptors. *Current Opinion in Immunology*.

[152] Kawai T, Akira S (2006). TLR signaling. *Cell Death and Differentiation*.

[153] Schofield L, Hackett F (1993). Signal transduction in host cells by a glycosylphosphatidylinositol toxin of malaria parasites. *Journal of Experimental Medicine*.

[154] Tachado SD, Gerold P, McConville MJ (1996). Glycosylphosphatidylinositol toxin of Plasmodium induces nitric oxide synthase expression in macrophages and vascular endothelial cells by a protein tyrosine kinase-dependent and protein kinase C-dependent signaling pathway. *Journal of Immunology*.

[155] Wu X, Gowda NM, Kumar S, Gowda DC (2010). Protein-DNA complex is the exclusive malaria parasite component that activates dendritic cells and triggers innate immune responses. *The Journal of Immunology*.

[156] Pichyangkul S, Yongvanitchit K, Kum-Arb U (2004). Malaria blood stage parasites activate human plasmacytoid dendritic cells and murine dendritic cells through a toll-like receptor 9-dependent pathway. *Journal of Immunology*.

[157] Hempelmann E, J. Egan T (2002). Pigment biocrystallization in Plasmodium falciparum. *Trends in Parasitology*.

[158] Franklin BS, Parroche P, Ataíde MA (2009). Malaria primes the innate immune response due to interferon-*γ* induced enhancement of toll-like receptor expression and function. *Proceedings of the National Academy of Sciences of the United States of America*.

[159] Jeney V, Balla J, Yachie A (2002). Pro-oxidant and cytotoxic effects of circulating heme. *Blood*.

[160] Balla J, Balla G, Jeney V, Kakuk G, Jacob HS, Vercellotti GM (2000). Ferriporphyrins and endothelium: a 2-edged sword—promotion of oxidation and induction of cytoprotectants. *Blood*.

[161] Balla J, Jacob HS, Balla G, Nath K, Eaton JW, Vercellotti GM (1993). Endothelial-cell heme uptake from heme proteins: induction of sensitization and desensitization to oxidant damage. *Proceedings of the National Academy of Sciences of the United States of America*.

[162] Seixas E, Gozzelino R, Chora Â (2009). Heme oxygenase-1 affords protection against noncerebral forms of severe malaria. *Proceedings of the National Academy of Sciences of the United States of America*.

[163] Arruda MA, Rossi AG, De Freitas MS, Barja-Fidalgo C, Graça-Souza AV (2004). Heme inhibits human neutrophil apoptosis: involvement of phosphoinositide 3-kinase, MAPK, and NF-*κ*B. *Journal of Immunology*.

[164] Graça-Souza AV, Arruda MAB, de Freitas MS, Barja-Fidalgo C, Oliveira PL (2002). Neutrophil activation by heme: implications for inflammatory processes. *Blood*.

[165] Porto BN, Alves LS, Fernández PL (2007). Heme induces neutrophil migration and reactive oxygen species generation through signaling pathways characteristic of chemotactic receptors. *Journal of Biological Chemistry*.

[166] Nomura S, Tandon NN, Nakamura T, Cone J, Fukuhara S, Kambayashi J (2001). High-shear-stress-induced activation of platelets and microparticles enhances expression of cell adhesion molecules in THP-1 and endothelial cells. *Atherosclerosis*.

[167] Mause SF, von Hundelshausen P, Zernecke A, Koenen RR, Weber C (2005). Platelet microparticles: a transcellular delivery system for RANTES promoting monocyte recruitment on endothelium. *Arteriosclerosis, Thrombosis, and Vascular Biology*.

[168] Amabile N, Guérin AP, Leroyer A (2005). Circulating endothelial microparticles are associated with vascular dysfunction in patients with end-stage renal failure. *Journal of the American Society of Nephrology*.

[169] Boulanger CM, Scoazec A, Ebrahimian T (2001). Circulating microparticles from patients with myocardial infarction cause endothelial dysfunction. *Circulation*.

[170] Fritzsching B, Schwer B, Kartenbeck J, Pedal A, Horejsi V, Ott M (2002). Release and intercellular transfer of cell surface CD81 via microparticles. *Journal of Immunology*.

[171] Baj-Krzyworzeka M, Szatanek R, Wȩglarczyk K (2006). Tumour-derived microvesicles carry several surface determinants and mRNA of tumour cells and transfer some of these determinants to monocytes. *Cancer Immunology, Immunotherapy*.

[172] Combes V, Coltel N, Alibert M (2005). ABCA1 gene deletion protects against cerebral malaria: potential pathogenic role of microparticles in neuropathology. *The American Journal of Pathology*.

[173] Khan ZM, Ng C, Vanderberg JP (1992). Early hepatic stages of *Plasmodium berghei*: release of circumsporozoite protein and host cellular inflammatory response. *Infection and Immunity*.

[174] Epiphanio S, Mikolajczak SA, Gonçalves LA (2008). Heme oxygenase-1 is an anti-inflammatory host factor that promotes murine plasmodium liver Infection. *Cell Host and Microbe*.

[175] De Weerd NA, Nguyen T (2012). The interferons and their receptors—distribution and regulation. *Immunology and Cell Biology*.

[176] de Veer MJ, Holko M, Frevel M (2001). Functional classification of interferon-stimulated genes identified using microarrays. *Journal of Leukocyte Biology*.

[177] Trinchieri G (2010). Type I interferon: friend or foe?. *Journal of Experimental Medicine*.

[178] Hisamatsu H, Shimbara N, Saito Y (1996). Newly identified pair of proteasomal subunits regulated reciprocally by interferon *γ*. *Journal of Experimental Medicine*.

[179] Boes B, Hengel H, Ruppert T, Multhaup G, Koszinowski UH, Kloetzel P (1994). Interferon *γ* stimulation modulates the proteolytic activity and cleavage site preference of 20S mouse proteasomes. *The Journal of Experimental Medicine*.

[180] Cramer LA, Nelson SL, Klemsz MJ (2000). Synergistic induction of the Tap-1 gene by IFN-*γ* and lipopolysaccharide in macrophages is regulated by STAT1. *The Journal of Immunology*.

[181] Wallach D, Fellous M, Revel M (1982). Preferential effect of *γ* interferon on the synthesis of HLA antigens and their mRNAs in human cells. *Nature*.

[182] Erbe DV, Collins JE, Shen L, Graziano RF, Fanger MW (1990). The effect of cytokines on the expression and function of Fc receptors for IgG on human myeloid cells. *Molecular Immunology*.

[183] Strunk RC, Cole FS, Perlmutter DH, Colten HR (1985). *γ*-Interferon increases expression of class III complement genes C2 and factor B in human monocytes and in murine fibroblasts transfected with human C2 and factor B genes. *Journal of Biological Chemistry*.

[184] Drevets DA, Leenen PJM, Campbell PA (1996). Complement receptor type 3 mediates phagocytosis and killing of Listeria monocytogenes by a TNF-*α*- and IFN-*γ*-stimulated macrophage precursor hybrid. *Cellular Immunology*.

[185] Yoshida A, Koide Y, Uchijima M, Yoshida TO (1994). IFN-*γ* induces IL-12 mRNA expression by a murine macrophage cell line, J774. *Biochemical and Biophysical Research Communications*.

[186] Gil MP, Bohn E, O'Guin AK (2001). Biologic consequences of Stat1-independent IFN signaling. *Proceedings of the National Academy of Sciences of the United States of America*.

[187] Taub DD, Lloyd AR, Conlon K (1993). Recombinant human interferon-inducible protein 10 is a chemoattractant for human monocytes and T lymphocytes and promotes T cell adhesion to endothelial cells. *The Journal of Experimental Medicine*.

[188] Ferreira A, Schofield L, Enea V (1986). Inhibition of development of exoerythrocytic froms of malaria parasites by *γ*-interferon. *Science*.

[189] Schofield L, Ferreira A, Altszuler R, Nussenzweig V, Nussenzweig RS (1987). Interferon-*γ* inhibits the intrahepatocytic development of malaria parasites in vitro. *The Journal of Immunology*.

[190] Mellouk S, Green SJ, Nacy CA, Hoffman SL (1991). IFN-*γ* inhibits development of Plasmodium berghei exoerythrocytic stages in hepatocytes by an L-arginine-dependent effector mechanism. *Journal of Immunology*.

[191] Nussler AK, Renia L, Pasquetto V, Miltgen F, Matile H, Mazier D (1993). In vivo induction of the nitric oxide pathway in hepatocytes after injection with irradiated malaria sporozoites, malaria blood parasites or adjuvants. *European Journal of Immunology*.

[192] Luty AJF, Lell B, Schmidt-Ott R (1999). Interferon-*γ* responses are associated with resistance to reinfection with Plasmodium falciparum in young African children. *Journal of Infectious Diseases*.

[193] Perlaza B, Sauzet J, Brahimi K, Benmohamed L, Druilhe P (2011). Interferon-*γ*, a valuable surrogate marker of Plasmodium falciparum pre-erythrocytic stages protective immunity. *Malaria Journal*.

[194] Schofield L, Villaquiran J, Ferreira A, Schellekens H, Nussenzweig R, Nussenzweig V (1987). Gamma Interferon, CD8+ T cells and antibodies required for immunity to malaria sporozoites. *Nature*.

[195] Stevenson MM, Tam MF, Belosevic M, van der Meide PH, Podoba JE (1990). Role of endogenous gamma interferon in host response to infection with blood-stage Plasmodium chabaudi AS. *Infection and Immunity*.

[196] Yoneto T, Yoshimoto T, Wang C (1999). Gamma interferon production is critical for protective immunity to infection with blood-stage Plasmodium berghei XAT but neither NO production nor NK cell activation is critical. *Infection and Immunity*.

[197] D'Ombrain MC, Robinson LJ, Stanisic DI (2008). Association of early interferon-*γ* production with immunity to clinical malaria: a longitudinal study among Papua New Guinean children. *Clinical Infectious Diseases*.

[198] Inoue S, Niikura M, Mineo S, Kobayashi F (2013). Roles of IFN-gamma and gammadelta T Cells in protective immunity against blood-stage malaria. *Frontiers in Immunology*.

[199] Luty AJF, Perkins DJ, Lell B (2000). Low interleukin-12 activity in severe Plasmodium falciparum malaria. *Infection and Immunity*.

[200] Aucan C, Walley AJ, Hennig BJW (2003). Interferon-alpha receptor-1 (IFNAR1) variants are associated with protection against cerebral malaria in The Gambia. *Genes & Immunity*.

[201] Simmons DP, Wearsch PA, Canaday DH (2012). Type I IFN drives a distinctive dendritic cell maturation phenotype that allows continued class II MHC synthesis and antigen processing. *Journal of Immunology*.

[202] Curtsinger JM, Valenzuela JO, Agarwal P, Lins D, Mescher MF (2005). Cutting edge: type I IFNs provide a third signal to CD8 T cells to stimulate clonal expansion and differentiation. *Journal of Immunology*.

[203] Agarwal P, Raghavan A, Nandiwada SL (2009). Gene regulation and chromatin remodeling by IL-12 and type I IFN in programming for CD8 T cell effector function and memory. *Journal of Immunology*.

[204] Hunter CA, Gabriel KE, Radzanowski T, Neyer LE, Remington JS (1997). Type I interferons enhance production of IFN-*γ* by NK cells. *Immunology Letters*.

[205] Gough DJ, Messina NL, Hii L (2010). Functional crosstalk between type I and II interferon through the regulated expression of STAT1. *PLoS Biology*.

[206] Jahiel RI, Vilcek J, Nussenzweig RS (1970). Exogenous interferon protects mice against Plasmodium berghei malaria. *Nature*.

[207] Van Damme J, Schaafsma MR, Fibbe WE, Falkenburg JHF, Opdenakker G, Billiau A (1989). Simultaneous production of interleukin 6, interferon-*β* and colony-stimulating activity by fibroblasts after viral and bacterial infection. *European Journal of Immunology*.

[208] Nussler A, Drapier J-ac, Renia L (1991). L-Arginine-dependent destruction of intrahepatic malaria parasites in response to tumor necrosis factor and/or interleukin 6 stimulation. *European Journal of Immunology*.

[209] Landau I, Gautret P, Sherman IW (1998). Animal models: rodents. *Malaria: Parasite Biology, Pathogenesis, and Protection*.

[210] Vigário AM, Belnoue E, Grüner AC (2007). Recombinant human IFN-*α* inhibits cerebral malaria and reduces parasite burden in mice. *Journal of Immunology*.

[211] Krupka M, Seydel K, Feintuch CM (2012). Mild Plasmodium falciparum malaria following an episode of severe malaria is associated with induction of the interferon pathway in Malawian children. *Infection and Immunity*.

[212] Taniguchi T, Ogasawara K, Takaoka A, Tanaka N (2001). IRF family of transcription factors as regulators of host defense. *Annual Review of Immunology*.

[213] Ozato K, Tailor P, Kubota T (2007). The interferon regulatory factor family in host defense: mechanism of action. *Journal of Biological Chemistry*.

[214] Kröger A, Köster M, Schroeder K, Hauser H, Mueller PP (2002). Activities of IRF-1. *Journal of Interferon and Cytokine Research*.

[215] Honda K, Takaoka A, Taniguchi T (2006). Type I interferon [corrected] gene induction by the interferon regulatory factor family of transcription factors. *Immunity*.

[216] Rihet P, Traoré Y, Abel L, Aucan C, Traoré-Leroux T, Fumoux F (1998). Malaria in humans: plasmodium falciparum blood infection levels are linked to chromosome 5q31-q33. *American Journal of Human Genetics*.

[217] Honda K, Yanai H, Takaoka A, Taniguchi T (2005). Regulation of the type I IFN induction: a current view. *International Immunology*.

[218] Liu Y, Zeng L, Tian AT (2012). Endoplasmic reticulum stress regulates the innate immunity critical transcription factor IRF3. *Journal of Immunology*.

[219] Yoneyama M, Suhara W, Fukuhara Y, Fukuda M, Nishida E, Fujita T (1998). Direct triggering of the type I interferon system by virus infection: activation of a transcription factor complex containing IRF-3 and CBP/p300. *EMBO Journal*.

[220] Wathelet MG, Lin CH, Parekh BS, Ronco LV, Howley PM, Maniatis T (1998). Virus infection induces the assembly of coordinately activated transcription factors on the IFN-*β* enhancer in vivo. *Molecular Cell*.

[221] Yie J, Senger K, Thanos D (1999). Mechanism by which the IFN-*β* enhanceosome activates transcription. *Proceedings of the National Academy of Sciences of the United States of America*.

[222] Sato M, Hata N, Asagiri M, Nakaya T, Taniguchi T, Tanaka N (1998). Positive feedback regulation of type I IFN genes by the IFN-inducible transcription factor IRF-7. *FEBS Letters*.

[223] Noah DL, Blum MA, Sherry B (1999). Interferon regulatory factor 3 is required for viral induction of beta interferon in primary cardiac myocyte cultures. *Journal of Virology*.

[224] Basler CF, Mikulasova A, Martinez-Sobrido L (2003). The Ebola virus VP35 protein inhibits activation of interferon regulatory factor 3. *Journal of Virology*.

[225] Ye J, Maniatis T (2011). Negative regulation of interferon-*β* gene expression during acute and persistent virus infections. *PLoS ONE*.

[226] Sagong M, Lee C (2011). Porcine reproductive and respiratory syndrome virus nucleocapsid protein modulates interferon-*β* production by inhibiting IRF3 activation in immortalized porcine alveolar macrophages. *Archives of Virology*.

[227] Tamura T, Ozato K (2002). ICSBP/IRF-8: its regulatory roles in the development of myeloid cells. *Journal of Interferon and Cytokine Research*.

[228] Horiuchi M, Itoh A, Pleasure D, Ozato K, Itoh T (2011). Cooperative contributions of interferon regulatory factor 1 (IRF1) and IRF8 to interferon-*γ*-mediated cytotoxic effects on oligodendroglial progenitor cells. *Journal of Neuroinflammation*.

[229] Driggers PH, Ennist DL, Gleason SL (1990). An interferon *γ*-regulated protein that binds the interferon- inducible enhancer element of major histocompatibility complex class I genes. *Proceedings of the National Academy of Sciences of the United States of America*.

[230] Nitcheu J, Bonduelle O, Combadiere C (2003). Perforin-dependent brain-infiltrating cytotoxic CD8^+^ T lymphocytes mediate experimental cerebral malaria pathogenesis. *Journal of Immunology*.

[231] Suidan GL, Mcdole JR, Chen Y, Pirko I, Johnson AJ (2008). Induction of blood brain barrier tight junction protein alterations by CD8 T cells. *PLoS ONE*.

[232] Fauconnier M, Palomo J, Bourigault ML (2012). IL-12R*β*2 is essential for the development of experimental cerebral malaria. *The Journal of Immunology*.

[233] Barnes BJ, Moore PA, Pitha PM (2001). Virus-specific activation of a novel Interferon regulatory factor, IRF-5, results in the induction of distinct interferon alpha genes. *The Journal of Biological Chemistry*.

[234] Barnes BJ, Kellum MJ, Field AE, Pitha PM (2002). Multiple regulatory domains of IRF-5 control activation, cellular localization, and induction of chemokines that mediate recruitment of T lymphocytes. *Molecular and Cellular Biology*.

[235] Fu XY, Kessler DS, Veals SA, Levy DE, Darnell JE (1990). ISGF3, the transcriptional activator induced by interferon *α*, consists of multiple interacting polypeptide chains. *Proceedings of the National Academy of Sciences of the United States of America*.

[236] Qureshi SA, Salditt-Georgieff M, Darnell JE (1995). Tyrosine-phosphorylated Stat1 and Stat2 plus a 48-kDa protein all contact DNA in forming interferon-stimulated-gene factor 3. *Proceedings of the National Academy of Sciences of the United States of America*.

[237] Sutherland CJ, Alloueche A, Curtis J (2002). Gambian children successfully treated with chloroquine can harbor and transmit *Plasmodium falciparum* gametocytes carrying resistance genes. *The American Journal of Tropical Medicine and Hygiene*.

[238] Na-Bangchang K, Muhamad P, Ruaengweerayut R, Chaijaroenkul W, Karbwang J (2013). Identification of resistance of *Plasmodium falciparum* to artesunate-mefloquine combination in an area along the Thai-Myanmar border: integration of clinico-parasitological response, systemic drug exposure, and *in vitro* parasite sensitivity. *Malaria Journal*.

[239] Hadden J, Friedman H, Klein T, Yamaguchi H (1992). Recent thoughts on the immunotherapy of infectious diseases including HIV infection. *Microbial Infections*.

[240] Jeffrey M, Jacobson M, Georgiev VS (2002). Immunotherapy for infectious diseases. *Infectious Disease*.

[241] Kak V, Sundareshan V, Modi J, Khardori NM (2012). Immunotherapies in infectious diseases. *Medical Clinics of North America*.

[242] Talpaz M, Kantarjian HM, McCredie K, Trujillo JM, Keating MJ, Gutterman JU (1986). Hematologic remission and cytogenetic improvement induced by recombinant human interferon alpha(A) in chronic myelogenous leukemia. *The New England Journal of Medicine*.

[243] Goldstein D, Laszlo J (1988). The role of interferon in cancer therapy: a current perspective. *Ca: A Cancer Journal for Clinicians*.

[244] Kirkwood JM, Ernstoff MS, Davis CA, Reiss M, Ferraresi R, Rudnick SA (1985). Comparison of intramuscular and intravenous recombinant alpha-2 interferon in melanoma and other cancers. *Annals of Internal Medicine*.

[245] Repetto L, Giannessi PG, Campora E (1996). Tamoxifen and interferon-beta for the treatment of metastatic breast cancer. *Breast Cancer Research and Treatment*.

[246] Windbichler GH, Hausmaninger H, Stummvoll W (2000). Interferon-gamma in the first-line therapy of ovarian cancer: a randomized phase III trial. *British Journal of Cancer*.

[247] Shiffman ML (2001). Pegylated interferons: what role will they play in the treatment of chronic hepatitis C?. *Current Gastroenterology Reports*.

[248] Chander G, Sulkowski MS, Jenckes MW (2002). Treatment of chronic hepatitis C: a systematic review. *Hepatology*.

[249] Yang J, Pu YG, Zeng ZM (2009). Interferon for the treatment of genital warts: a systematic review. *BMC Infectious Diseases*.

[250] Achtman AH, Pilat S, Law CW (2012). Effective adjunctive therapy by an innate defense regulatory peptide in a preclinical model of severe malaria. *Science Translational Medicine*.

